# Oncogenic kinase fusions: an evolving arena with innovative clinical opportunities

**DOI:** 10.18632/oncotarget.7853

**Published:** 2016-03-02

**Authors:** Fabrizio Tabbò, Marco Pizzi, Peter W. Kyriakides, Bruce Ruggeri, Giorgio Inghirami

**Affiliations:** ^1^ Department of Molecular Biotechnology and Health Science and Center for Experimental Research and Medical Studies (CeRMS), University of Torino, Torino, Italy; ^2^ Department of Pathology and Laboratory Medicine, Weill Cornell Medical College, New York, NY, USA; ^3^ General Pathology and Cytopathology Unit, Department of Medicine-DIMED, University of Padova, Padova, Italy; ^4^ Pre-Clinical Discovery Biology, Incyte Corporation, Wilmington, DE, USA; ^5^ Department of Pathology, and NYU Cancer Center, New York University School of Medicine, New York, NY, USA

**Keywords:** tyrosine kinase fusions, translocations, signaling pathways, small molecule inhibitors, resistance mechanisms

## Abstract

Cancer biology relies on intrinsic and extrinsic deregulated pathways, involving a plethora of intra-cellular and extra-cellular components. Tyrosine kinases are frequently deregulated genes, whose aberrant expression is often caused by major cytogenetic events (e.g. chromosomal translocations). The resulting tyrosine kinase fusions (TKFs) prompt the activation of oncogenic pathways, determining the biological and clinical features of the associated tumors. First reported half a century ago, oncogenic TKFs are now found in a large series of hematologic and solid tumors. The molecular basis of TKFs has been thoroughly investigated and tailored therapies against recurrent TKFs have recently been developed. This review illustrates the biology of oncogenic TKFs and their role in solid as well as hematological malignancies. We also address the therapeutic implications of TKFs and the many open issues concerning their clinical impact.

## INTRODUCTION

The discovery of Philadelphia chromosome (Ph) in Chronic Myeloid Leukemia (CML) [1], the subsequent identification of the genes involved in this translocation [2] and the description of its molecular mechanisms [3, 4] have marked a scientific milestone, which dramatically shaped our understanding of kinase-mediated oncogenesis.

Since these findings, many studies have investigated cell transformation via Tyrosine Kinase Fusions (TKFs). A recent appraisal of the literature shows that ~1000 independent studies have been published describing the oncogenic properties of TKFs. Recurrent kinase chimeras are detected in ~2-3% of human tumors and this frequency increases when non-recurrent fusions (4-5%) or cancer-associated abnormal karyotypes (5-7%) are considered [5]. TKFs do not represent the only cancer-associated gene derangement involving Tyrosine Kinases (TKs). During oncogenic transformation, TKs can indeed undergo a number of molecular impairments, including: (i) gene mutations; (ii) gene amplifications; (iii) chromosomal translocations; and (iv) non-fusion rearrangements (e.g. CRLF2 rearrangements in ALL) [6, 7]. All such Tyrosine Kinase (TK)-associated aberrations induce a huge variety of metabolic and signaling derangements, which ultimately lead to neoplastic transformation.

The widespread occurrence of TKFs in human cancers, their ability to synergistically fire multiple signaling pathways and their overall pivotal role in clinical oncology justify the many recent efforts to identify new anti-TKF target therapies. Of note, similar TKF-related pathways have been reported in different tumors; this constitutes the rationale for the use of similar therapies in tumors bearing the same molecular derangements. This approach is well represented by Ph-like leukemias, whose transcriptomes closely resemble those of conventional Ph-positive ALL, even in the absence of BCR-ABL translocations [8, 9]. This model and its corresponding Patient Derived Tumor Xenografts (PDTX) allow the assessment of therapeutic agents targeting common molecular “hubs” [10]. This perspective is in line with the new “basket trial” era, where patients with different tumor types may be grouped together based on their tumors' mutational profile.

In this review, the role of TKFs in both hematologic and solid neoplasms will be discussed. Special attention will be devoted to the many open issues concerning the treatment of TKF-bearing neoplasms.

## STRUCTURES OF TKFS AND ROLE OF THE N-TERMINUS DOMAINS

Tyrosine kinases are the most common kinases and catalyze the transfer of a phosphate group from ATP to tyrosine residues. On a structural level, TK display a variety of modules dictating their cellular localization, function stability and response to therapy [11]. The enzymatic activity of these proteins relies on their TK domains (TKD), which are targeted by ATP-competitive and irreversible, covalent inhibitors [12]. Other important regions are the SH2 domains, the extracellular domains and remote allosteric sites, which can also be targeted by neutralizing drugs [13].

**Figure 1:**
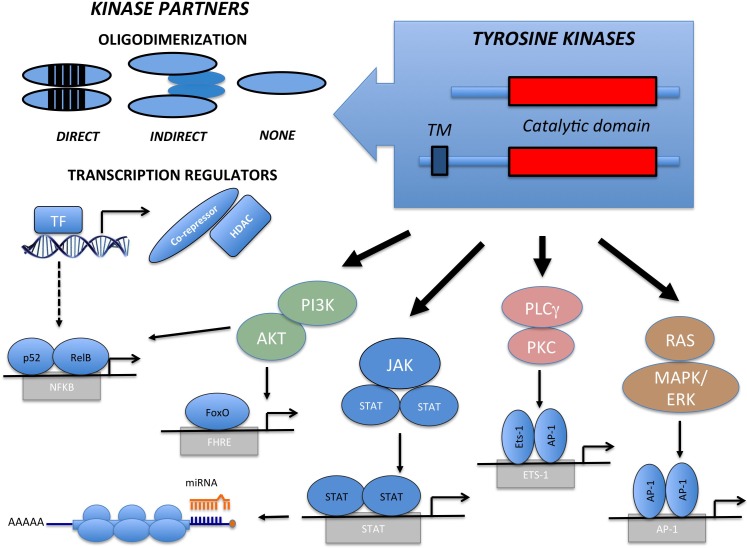
Structure and Signaling Transduction Motifs of Tyrosine Kinase Fusions The constitutive activation of Tyrosine Kinase Fusion oncoproteins are achieved through multiple mechanisms taking advantage of direct or indirect oligodimerization. Seldom no dimerization are required. Fusion partners can also engage *per se* oncogenic signaling pathways, directly or indirectly modulating Transcription Factors (i.e. NFkB) and their corresponding genes. Kinase activation induces multiple canonical pathways (PI3K/AKT, JAK/STAT, PLCγ/PKC and RAS/ERK), which regulate genes controlling transcription and providing pro-tumorigenic signals. Compensatory pathways and regulatory modalities may act in place (i.e. miRNA regulation).

TKD are highly conserved modules, frequently composed by a N-terminal ATP-binding region and by a regulatory C-terminal domain. Upon activation, a cascade of events occurs, including ATP consumption, kinase dimerization, conformational changes, trans-autophosphorylation and oligo-complex formation. TK regulation is disrupted in oncogenic TKFs, which display constitutive dimerization due to loss of the inhibitory domains and forced oligodimerization of partner peptides [14]. Both inter- and intra-chromosomal rearrangements can lead to TKFs. Most break-points occur within introns and allow the synthesis of chimeric transcripts carrying the functional kinase domain and its flanking regions (Tables 1 and 2). Translocations involving TK membrane receptors frequently have breakpoints between exons coding for the juxta-membrane region. Though less frequent, the breaks can also split the trans-membrane domain upstream from the GXGXXG-coding exon. If protein products presenting the TK transmembrane domain localize to the cellular membrane, the exclusion of such a domain will localize the TK according to the properties of the translocation partner instead (Figure 1). In both cases, the natural TK ligand-binding site is lost. TKF involving intra-cytoplasmic kinases are also characterized by the loss of regulatory regions.

**Table 1:** Tyrosine kinases fusion in hematological malignancies

Kinase	Activating Mechanisms	Chromosomal translocation	Entity	Kinase inhibitor	Frequency (%)	Reference
*ALK*	Fusion to NPM1	t(2;5)(p23;q35)	ALCL, DLBCL	Crizotinib	75-80, N/A	Morris *et al.* 1994, Adam *et al*. 2003
	Fusion to ALO17	t(2;17)(p23;q25)	ALCL	Crizotinib	<1	Cools *et al.* 2002
	Fusion to TGF	t(2;3)(p23;q21)	ALCL	Crizotinib	2	Hernandez *et al*. 1999
	Fusion to MSN	t(2;X)(p32;q11-12)	ALCL	Crizotinib	<1	Tort *et al.* 2001
	Fusion to TPM3	t(1;2)(q25;p23)	ALCL	Crizotinib	12-18	Lamant *et al*. 1999
	Fusion to TPM4	t(2;19)(p23;p13)	ALCL	Crizotinib	<1	Meech *et al*. 2001
	Fusion to ATIC	inv(2)(p23;q35)	ALCL	Crizotinib	2	Ma *et al.* 2000
	Fusion to MYH9	t(2;22)(p23;q11.2)	ALCL	Crizotinib	<1	Lamant *et al.* 2003
	Fusion to TRAF1	t(2;9)(p23;q33)	ALCL	Crizotinib	<1	Feldman AL *et al.* 2013
	Fusion to CLTC1	t(2;17)(p23;q23)	ALCL, DLBCL	Crizotinib	2, N/A	Touriol *et al.* 2000
	Fusion to SQSTM1	t(2;5)(p23.1;q35.3)	DLBCL	Crizotinib	N/A	Takeuchi *et al.* 2010
	Fusion to SEC31A	t(2;4)(p24;q21)	DLBCL	Crizotinib	N/A	Bedwell *et al.* 2007
	Fusion to RANBP2	inv(2)(p23;q13)	AML	Crizotinib	<1	Maesako *et al.* 2014
*ABL*	Fusion to BCR	t(9;22)(q34;q11)	CML, B-ALL	Imatinib	85-90, <30	Klein et al. 1982
	Fusion to TEL	t(9;12)(q34;p13)	AML	Dasatinib	<1	Golub *et al.* 1996
	Fusion to NUP214	t(9;9)(q34.1;q34.3)	T-ALL, Ph-like ALL	Nitolinib	5	Graux *et al.* 2004, Roberts *et al* 2014
	Fusion to EML1	t(9;14)(q34;q32)	T-ALL	Nitolinib	<1	De Keersmaecker *et al*. 2005
	Fusion to ZMIZ1	t(9;10)(q34;q22)	B-ALL	N/A	<1	Soler *et al.* 2008
	Fusion to RCSD1	t(1;9)(q24;q34)	B-ALL	Dasatinib	<1	Mustjoki *et al*. 2009
	Fusion to FOXP1	t(3;9)(p12;q34)	B-ALL	N/A	<1	Ernst T *et al.* 2011
	Fusion to SNX2	t(5;9)(q23;q34)	B-ALL	Imatinib	<1	Ernst T *et al.* 2011, Masuzawa *et al.* 2014
	Fusion to SEPT9	t(9;17)(q34;q25)	T-PLL	N/A	<1	Suzuki *et al.* 2014
	Fusion to multiple partners	t(9;12)(q34;p13)	Ph-like ALL	Dasatinib	<1	Roberts *et al.* 2012, Roberts *et al.* 2014
*ARG*	Fusion to TEL	t(1;12)(q25;p13)	AML, aCML	Imatinib	<1	Golub *et al.* 1995
*PDGFRa*	Fusion to FIP1L1	t(4;12)(q23;p12)	HES	Imatinib	12	Cools *et al*. 2003
	Fusion to BCR	t(4;22)(q12;q11.2)	CEL, T-ALL, CML-like MPN	Imatinib	<1	Baxter *et al.* 2002, Yigit *et al.* 2015, Cluzeau *et al.* 2015
	Fusion to TNKS2	t(4;10)(q12;q23.3)	MPN w/eosinophilia	Imatinib	<1	Chalmer *et al.* 2014
	Fusion to STRN	t(2;4)(p22;q12)	MPN w/eosinophilia	Imatinib	<1	Curtis *et al.* 2007
	Fusion to ETV6	t(4;12)(q23;p12)	MPN w/eosinophilia	Imatinib	<1	Curtis *et al.* 2007
	Fusion to KIF5B	t(4;10)(q12;p11)	MPN w/eosinophilia	Imatinib	<1	Score *et al.* 2006
	Fusion to CDK5RAP2	ins(9;4)(q33;q12q25)	CEL	Imatinib	<1	Walz *et al.* 2006
*PDGFRb*	Fusion to TEL	t(5;12)(q33;p13)	CMML	Imatinib	4	Golub *et al.* 1994
	Fusion to HIP1	t(5;7)(q33;q11)	CMML	Imatinib	4	Ross *et al.* 1998
	Fusion to Rabaptin5	t(5;17)(q33;p13)	CMML	Imatinib	<1	Magnusson *et al.* 2001
	Fusion to H4(D10S170)	t(5;10)(q33;q11-q21)	aCML	Imatinib	<1	Kulkarni *et al.* 2000
	Fusion to CEV14	t(5;14)(q33;q32)	AML	Imatinib	<1	Abe *et al.* 1997
	Fusion to Myomegalin	t(1;5)(q23;q33)	Eosinophilia	Imatinib	<1	Wilkinson *et al.* 2003
	Fusion to ATF71P	t(5;12)(q23;p13)	Ph-like ALL	Imatinib	<1	Kobyashi *et al.* 2015
	Fusion to EBF1	t(5;5)(q33.1;q33.3)	Ph-like ALL	Dasatinib	<1	Roberts *et al.* 2012, Roberts *et al.* 2014
*FGFR1*	Fusion to multiple partners	t(2;8)(q12;p11)	EMS, AML	None	<1	Etienne *et al.* 2007
	Fusion to FOP	t(6; 8)(q27;p11)	MPN	None	<1	Lee *et al.* 2014
	Fusion to SQSTM1	t(5;8)(q35;p11)	AML		<1	Nakamura Y *et al.* 2014
*FGFR3*	Fusion to TEL	t(4;12)(p16;p13)	PTCL	Fiin23, NVP-BGJ398	<1	Maeda *et al.* 2005
	Fusion to IGH	t(4;14) (p16; q32)	CLL		<1	Geller *et al.* 2014
	Fusion to TIF1	t(7;8)(q34;p11)	MDS, CLL, AML	Fiin23, NVP-BGJ398	<1	Maeda *et al.* 2005
*JAK2*	Fusion to TEL	t(9;12)(p24;p13)	ALL, CML-like	Ruxolitinib	<5	Lacronique *et al.* 1997
	Fusion to OFD1	t(X;9)(p22;p24)	ALL	Jak2 inhibitors	<1	Yano *et al.* 2015
	Fusion to SPAG9	t(9;17)(p24;q21)	ALL	Jak2 inhibitors	<1	Kavamura M *et al* 2015
	Fusion to PAX5	t(9;9)(p13;p24)	ALL	Jak2 inhibitors	<1	Nebral K *et al.* 2009
	Fusion to BCR	t(9;22)(p24;q11.2)	aCML	Ruxolitinib	<5	Griesinger *et al.* 2005
	Fusion to multiple partners	t(9;12)(p24;p13)	Ph-like ALL	Jak2 inhibitors	<1	Roberts *et al.* 2012, Roberts *et al.* 2014
*NTRK3*	Fusion to TEL	t(12;15)(p13;q25)	AML	None	<1	Knezevich *et al.* 1998
*SYK*	Fusion to TEL	t(9;12)(q22;p12)	MDS	Imatinib	<1	Kanie *et al.* 2004
	Fusion to ITK	t(5;9)(q33;q22)	PTCL-NOS, AITL	None	17, <1	Streubel B *et al* 2006, Attygale et al 2013
*TRKC*	Fusion to TEL	t(12;15)(p13;q25)	AML and fibrosarcome	None	<1	Dobus *et al.* 2001
*FLT3*	Fusion to ETV6	t(12;13)(p13;q12)	HES	Sunitinib, Midostaurin, Lestaurtinib	<1	Vu *et al.* 2006
*ETV6*	Fusion to TEL	t(4;12)(p16;p13)	PTCL-NOS	Ruxolitinib	<1	Yagasaki *et al.* 2001
*CSF1R*	Fusion to SSBP2	t(5;5)(q14;q33)	Ph-like ALL	Dasatinib	<1	Roberts *et al.* 2014
*CRLF2*	Fusion to IGH	t(X;14)(p22;q32)/t(Y;14)(p11;q32)	Ph-like ALL	Jak2 inhibitors	<5	Mullighan *et al.* 2009, Roberts *et al.* 2014
*ROS1*	Fusion to NFKB2	t(6;10)(q22;q24)	ALCL	Ros1 inhibitors	<1	Crescenzo *et al.* 2015
	Fusion to NCOR2	t(6;12)(q22;q24)	ALCL	Ros1 inhibitors	<1	Crescenzo *et al.* 2015
*TYK2*	Fusion to NFKB2	t(19;10)(p13;q24)	ALCL	Tyk2 inhibitors	<1	Crescenzo et al. 2015
	Fusion to NPM1	t(19;5)(p13;q35)	LPDs	Tyk2 inhibitors	<1	Velusamy *et al.* 2014
*LYN*	Fusion to NCOR	t(8;17)(q13;p11)	ALL	NA	<1	Yano *et al.* 2015

**Abbreviation:** ALCL: anaplastic large cell lymphoma, AML: acute myeloid leukemia, B-ALL: B-cell acute lymphoblastic leukemia; T-ALL: T-cell acute lymphoblastic leukemia, Ph-like ALL: Philadelphia Chromosome like acute lymphoblastic leukemia, CEL: chronic eosinophilic leukemia, CML: chronic myeloid leukaemia; aCML: atypical chronic myeloid leukemia, CMML: chronic myelomonocytic leukemia, DLBCL: diffuse large B-cell lymphoma, EMS: 8p11 myeloproliferative syndrome, HES: hyper eosinophilic syndrome, LPDs: lymphoproliferative disorders, MDS: myelodysplastic syndrome, MPN: myeloproliferative neoplasm, PTCL-NOS: peripheral T-cell lymphoma not otherwise specified

**Table 2:** Tyrosine kinases fusions human in solid tumors

Kinase	Activating Mechanisms	Chromosomal translocation	Entity	Kinase inhibitor	Frequency (%)	Reference
*ALK*	Fusion to ATIC	inv(2)(p23;q35)	IMT	Crizotinib	< 5	Debiec-Rychter *et al.* 2003
	Fusion to CARS	t(2;11)(p23;p15)	IMT	Crizotinib	<5	Cools *et al.* 2002
	Fusion to CLTC	t(2;17)(p23;q23)	IMT	Crizotinib	<5	Bridge *et al*. 2001
	Fusion to EML4	inv(2)(p21;p23)	NSCLC	Crizotinib, Ceritinib, Alecitinib	2-5	Soda *et al.* 2007
			BC, CRC	Crizotinib	<5	Lin *et al.* 2009
	Fusion to FN1	t(2;11)(q31;p15)	Soft tissue sarcoma	Crizotinib	2-4	Ren *et al.* 2012
	Fusion to KIF5B	t(2;10)(p23;p11)	NSCLC	Crizotinib	<1	Takeuchi *et al.* 2009
	Fusion to KLC1	t(2;14)(p23;q32)	NSCLC	Crizotinib	<5	Jung *et al.* 2012
	Fusion to RANBP2	t(2;2)(p23;q13)	IMT	Crizotinib	<5	Ma *et al.* 2003
	Fusion to SEC31L1	t(2;4)(p23;q21)	IMT	Crizotinib	<5	Panagopoulos *et al.* 2006
	Fusion to VCL	t(2;10)(p23;q22)	RCC	Crizotinib	<3	Debelenko *et al.* 2011
	Fusion to SEC31A	t(2;4)(p23;q21)	NSCLC	Crizotinib	<1	Kim *et al.* 2015
	Fusion to STRN	t(2;2)(p23;p22)	Thyroid cancer	Crizotinib	<1	Pérot *et al.* 2014, Kelly *et al.* 2013
			NSCLC	Crizotinib	<1	Majewski *et al.* 2013
	Fusion to GTF2IRD1	t(2;7)(p23;q11.23)	Thyroid cancer	Crizotinib	<1	Stransky et al. 2015
	Fusion to TFG	t(2;3)(p23;q21)	NSCLC	Crizotinib	2	Rikova *et al.* 2007
	Fusion to TPM1	t(2;15)(p23;q22.2)	Bladder cancer	Crizotinib	<1	Stransky *et al.* 2015
	Fusion to TPM3	t(1;2)(q21;p23)	IMT	Crizotinib	50	Lawrence *et al.* 2000
	Fusion to TPM4	t(2;19)(p23;p13)	IMT	Crizotinib	<5	Lawrence *et al.* 2000
	Fusion to PTPN3	t(2;9)(p23;q31.3)	NSCLC	Crizotinib	<1	Jung *et al.* 2012
	Fusion to A2M	t(2;12)(p23;p13)	FLIT	Crizotinib	<1	Onoda *et al.* 2014
	Fusion to TPR	t(2;1)(p23;q31.1)	NSCLC	Crizotinib	<1	Choi *et al.* 2014
	Fusion to HIP1	t(2;7)(p23;q11.23)	NSCLC	Crizotinib	<1	Hong *et al.* 2014
	Fusion to SQSTM1	t(2;5)(p23;q35)	NSCLC	Crizotinib	<1	Iyevleva *et al.* 2015
	Fusion to DCTN1	t(2;2)(p23;p13)	NSCLC	Crizotinib	<1	Iyevleva *et al.* 2015
	Fusion to SMEK2	t(2;2)(p23;p16.1)	CRC	Crizotinib	<1	Stransky et al. 2015
	Fusion to CAD	inv(2)(p22-21p23)	CRC	Entrectinib	<1	Lee *et al.* 2015, Amatu *et al.* 2015
*ROS1*	Fusion to CD74	t(5;6)(q32;q22)	NSCLC	Crizotinib	<2	Bergethon *et al.* 2012
	Fusion to EZR	inv(6)(q22q25.3)	NSCLC	Crizotinib	<2	Arai *et al.* 2013
	Fusion to GOPC	del(6)(q22q22.3)	NSCLC	Crizotinib	<2	Rimkunas *et al.* 2012, Suehara *et al.* 2012
			CCA	Crizotinib	<1	Gu *et al.* 2011
			Ovarian Cancer	Crizotinib	<1	Birch *et al.* 2011
	Fusion to LRIG3	t(6;12)(q22;q14.1)	NSCLC	Crizotinib	<2	Takeuchi *et al.* 2012
	Fusion to SDC4	t(6;20)(q22;q12)	NSCLC	Crizotinib	<2	Davies *et al.* 2012, Takeuchi *et al.* 2012
	Fusion to SLC34A2	t(4;6)(q15.2;q22)	NSCLC	Crizotinib	<2	Davies *et al.* 2012
			Gastric cancer	Crizotinib	<1	Lee *et al.* 2013
	Fusion to TPM3	t(1;6)(q21.2;q22)	NSCLC	Crizotinib	<2	Takeuchi *et al.* 2012
	Fusion to TFG	t(6;3)(q22.1;q12.2)	IMT	Crizotinib	<1	Yamamoto *et al.* 2015
*RET*	Fusion to CCDC6	inv10(q11;q21)	NSCLC	Cabozantinib, Vandetanib	<2	Wang *et al.* 2012
			Thyroid cancer	Cabozantinib, Vandetanib	<2	Celestino *et al.* 2012
	Fusion to KIF5B	inv(10)(p11;q11)	NSCLC	Cabozantinib, Vandetanib	<2	Ju *et al.* 2012
	Fusion to NCOA4	inv(10)(q11;q11)	Thyroid cancer	Cabozantinib, Vandetanib	<2	Rui *et al*. 2012
	Fusion to PRKAR1A	t(10;17)(q11.2;q23)	Thyroid cancer	Cabozantinib, Vandetanib	<2	Rui *et al*. 2012
	Fusion to ACBD5	inv(10)(p12.1;q11.2)	Thyroid cancer	Cabozantinib, Vandetanib	<1	Hamatani *et al.* 2014
*BRAF*	Fusion to KIAA1549	t(7;7)(q34;q34)	Brain tumors	BRAF/MEK inhibitors	<1	Tian *et al.* 2011
	Fusion to FAM131B	t(7;7)(q34;q34)	Brain tumors	BRAF/MEK inhibitors	<1	Cin *et al.* 2011
	Fusion to CEP89	t(7;19)(q34;q13)	Melanoma	BRAF/MEK inhibitors	< 5	Wiesner *et al.* 2014
	Fusion to LSM14A	t(7;19)(q34;q13)	Melanoma	BRAF/MEK inhibitors	< 5	Wiesner *et al.* 2014
*FGFR1*	Fusion to TACC1	t(8;8)(p11.23;p11.22)	GBM	FGFR inhibitor	<3	Singh *et al.* 2012
	Fusion to BAG4	t(8;8)(p11.23;p11.23)	NSCLC	FGFR inhibitor	<1	Rui et al. 2014
*FGFR2*	Fusion to BICC1	t(10;10)(q26;q21.1)	CCA	FGFR inhibitor	<1	Yi-Mi *et al.* 2013
	Fusion to KIAA1967	t(10;8)(q26;p21.3)	NSCLC	FGFR inhibitor	<1	Yi-Mi et al. 2013
	Fusion to PPHLN1	t(10;12)(q26;q12)	CCA	FGFR inhibitor	45	Sia *et al.* 2015
*FGFR3*	Fusion to TACC3	del4(p16;p16)	GBM	FGFR inhibitor	<3	Singh *et al.* 2012, Bao *et al.* 2014
			Bladder cancer	FGFR inhibitor	<2	Williams *et al.* 2013
			NSCLC	FGFR inhibitor	<2	Rui *et al.* 2014
			ESCC	FGFR inhibitor	<1	Yuan *et al.* 2014
			NPC	FGFR inhibitor	<3	Yuan *et al.* 2014
			Cervical cancer	FGFR inhibitor	<1	Carneiro *et al.* 2015
*NTRK1*	Fusion to TPM3	t(1;3)(q21;q11)	Thyroid cancer	TRKA inhibitor	7-8	Beimfohr *et al.* 1999
			CRC	TRKA inhibitor	<1	Creancier *et al.* 2015
			HGG	TRKA inhibitor	<1	Wu *et al.* 2014
	Fusion to TPR	inv1(q23;q21)	Thyroid cancer	TRKA inhibitor	<1	Greco *et al.* 1999
			CRC	TRKA inhibitor	<1	Creancier *et al.* 2015
	Fusion to MPRIP	t(1;17)(q21;p11)	NSCLC	TRKA inhibitor	<5	Vaishanvi *et al.* 2013
	Fusion to CD74	t(1;5)(q21;q32)	NSCLC	TRKA inhibitor	<5	Vaishanvi *et al.* 2013
	Fusion to RABGAP1L	t(1;1)(q21;q25.1)	CCA	TRKA inhibitor	<1	Ross *et al.* 2014
	Fusion to SQSTM1	t(1;5)(q21;q35)	NSCLC	Entrectinib	<1	Farago *et al.* 2015
	Fusion to LMNA	t(1;1)(q21;q22)	Soft tissue sarcoma	LOXO-101	<1	Doebele *et al.* 2015
			CRC	Entrectinib	<1	Sartore-Bianchi *et al.* 2015
*NTRK2*	Fusion to VCL	t(9;10)(q22.1;q22)	HGG	TRKA inhibitor	<1	Wu et al. 2014
	Fusion to AGBL4	t(9;1)(q22.1;p33)	HGG	TRKA inhibitor	<1	Wu et al. 2014
*NTRK3*	Fusion to ETV6	t(12;15)(p13;q25)	Thyroid cancer	TRKA inhibitor	2-14	Ricarte-Filho *et al.* 2013
			CFS	TRKA inhibitor	<1	Knezevih *et al.* 1998
			IMT	TRKA inhibitor	<1	Yamamoto *et al.* 2015
			GIST	TRKA inhibitor	<1	Brenca *et al.* 2015
			MASC	TRKA inhibitor	<1	Skalovà *et al.* 2015
			HGG	TRKA inhibitor	<1	Wu et al. 2014
	Fusion to BTBD1	t(15;15)(p24;q25)	HGG	TRKA inhibitor	<1	Wu et al. 2014
*AKT2*	Fusion to BCAM	t(19;19)(q13.2;q13.3)	HGSC	AKT2 inhibitor	10	Kannan *et al.* 2015
*PRKACA*	Fusion to DNAJB1	t(19;19)(p13.1;p13.2)	FL-HCC	PKA inhibitors	100	Honeyman *et al.* 2014
*PRKD1*	Fusion to ARID1A	t(14;1)(q12;p36)	Salivary gland tumor	PRKD1 inhibitor	<3	Weinreb *et al.* 2014
*MET*	Fusion to PTPRZ1	t(7;7)(q31.2;q31.3)	GBM	MET inhibitor	15	Bao *et al.* 2014
*PIK3CA*	Fusion to TBL1XR1	t(3;3)(q26.3;q26.32)	BC	PIK3CA inhibitor	<1	Stransky et al. 2015

**Abbreviation:** BC: breast cancer, CCA: cholangiocarcinoma, CFS: congenital fibrosarcoma, CRC: colon-rectal cancer, ESCC: esophageal squamous cell carcinoma, FL-HCC: fibrolamellar hepatocellular carcinoma, FLIT: fetal lung interstitial tumor, GBM: glioblastoma, GIST: gastrointestinal stromal tumor, HGG: high grade glioma, HGSC: high-grade serous ovarian cancer, IMT: imflammatory myofibroblatic tumor, MASC: mammary analogue secretory carcinoma of salivary glands, NPC: nasopharyngeal carcinoma, NSCLC: non-small cell lung cancer

Gene fusions typically replace the TK promoter; therefore TKF expression becomes ectopically regulated by the promoter of the partner gene. Partner genes contribute to the oncogenic potential in a number of ways. In most instances, the partner N-terminus region provides dimerization domains, which recruit molecular adaptors and lead to the constitutive trans-phosphorylation and activation of the kinase. TKF partners can also directly activate oncogenic pathways, as observed in the TFG-NTRK1 and TRAF1-ALK fusions, independently activating the NF-kB pathway [15]. We and others have recently demonstrated that a small subset of systemic (<5%) and cutaneous (~20%) Anaplastic Large Cell Lymphomas (ALCL) bears ROS1 or TYK2 fusions. In such cases, the TKF N-terminus region comprises a transcription factor (NFkB) or a transcriptional repressor (NCOR2) fused to ROS1 and TYK2, respectively [16]. These TKFs clearly illustrate how the chimera may also influence the transcriptional activity of the partner genes, perturbing transcription promotion or repression. This, in turn, may lead to a widespread derangement of several metabolic and signaling pathways, which ultimately concur to neoplastic transformation [15, 16]. The N-terminus can also contribute to the neoplastic phenotype by preventing protein degradation through the recruitment of heat shock proteins (Hsp70, Hsp90) [17]. This may constitute a valid therapeutic target, as observed with Hsp90 inhibitors for the treatment of NPM-ALK ALCL [16, 17] and FLT3-positive leukemias [18]. Fusion partners can further contribute to neoplastic transformation by directing TKF localization. In T-ALL, for example, NUP214 of the NUP214-ABL orchestrates TKF nuclear pore localization, which is required for neoplastic transformation [19]. Similarly, the ectopic localization of NPM-ALK and FOP-FGFR1 contributes to oncogenesis by impacting centrosome deregulation and chromosomal instability. Lastly, fusion partners can act as dominant negative mutants. By decreasing the access to wild-type TPM3, TPM3-ALK fusions induce changes in the cytoskeleton organization and confer a higher metastatic capacity [20].

## TKF IN HUMAN CANCERS

TKFs are found in many human cancers, although at very different frequencies. Among recurrent translocations, hematologic cancers were first shown to carry rearrangements characterized by TKF expression. Subsequent studies demonstrated that *RET* [21], *TRK* [22] or *NTRK1* [23] were frequently translocated in a fraction of thyroid carcinomas, as well as other epithelial and soft tissue neoplasms [24, 25]. This list has grown steadily to >300 oncogenic TKF [5] (http://cgap.nci.nih.gov/Chromosomes/Mitelman) (Tables 1 and 2).

### Are TKF drivers or passengers in human oncogenesis?

There is strong evidence that both TK and TKFs play an oncogenic role in human cancers [26-28]. Nevertheless, TKF-driven transformation requires multiple genomic defects and the contribution of the host microenvironment to drive oncogenesis. This paradigm is well represented by Chronic Eosinophilic Leukemia (CEL), where the sustained expression of FIP1L1-PDGFRA is necessary (but insufficient) to sustain eosinophils proliferation [29]. In humans, CEL also requires the overexpression of IL-5 through a single nucleotide polymorphism of *IL5RA*. The same holds true for systemic mastocytosis (SM), in which the constitutive activation of c-KIT alone is insufficient to induce the neoplastic phenotype and requires the stimulation of stem cell factor (SCF) as well [30].

This view is somehow challenged by seminal studies on other myeloproliferative neoplasms (MPNs), like CML, which may in fact represent monogenic disorders. The implantation of BCR-ABL (p210) retroviral-transduced marrow precursors into recipient mice can indeed cause rapidly progressing MPNs, which closely resemble human CML [31]. Of note, this scenario is hardly achievable in mouse models, where the TKF is ectopically expressed by means of more conventional molecular strategies [32, 33]. Moreover, Foley *et al.* have recently shown that the expression of BCR-ABL alone from the knock-in allele is unable to induce leukemia [34], suggesting that this TKF is *per se* insufficient to induce a disease fully recapitulating human CML. This latter hypothesis is consistent with the observation of BCR-ABL-positive hematopoietic cells also in healthy individuals [35], but is challenged by the observation that a fraction of CML patients who have achieved a sustained complete molecular remission (CMR) on imatinib can remain in CMR after drug withdrawal [36]. This latter observation raises the possibility that BCR-ABL signaling may not be the only driving force in some cases of CML. Of note, the concomitant loss of p14 (ARF) [37-39], and the critical role of Bcl-6 [40], miR-29 [41], and Raf1 [42] in CML suggest that the co-occurrence of selective defects and/or the full activation of selective pathways are required to achieve the complete neoplastic phenotype. This is line with the recent observation that IKF1-mutated Ph-positive high-risk ALL, in which IKF1 and Arf alterations synergistically promote the development of an aggressive lymphoid leukemia, can be effectively treated with retinoid receptor agonists which potentiate the activity of dasatinib in mouse and human BCR-ABL1 ALL [43].

The evidence that normal T-lymphocytes can undergo neoplastic transformation upon ectopic expression of fusion proteins alone (e.g. NPM-ALK) may further challenge the aforementioned paradigm [44]. Further studies are however needed to clarify this issue, as TKF transcripts have also been documented in healthy T-cells and in normal hematopoietic cells [35, 45]. Taken together, these observations indicate that TKFs alone are probably unable to drive T-cell neoplastic transformation completely and require secondary chromosomal abnormalities/TK activating mutations [46].

TKF expression levels may also play a role in tumor development. This is the case of ALK-translocated tumors: high NPM-ALK expression is indeed seen in ALCL, while lower EML4-ALK levels characterize NSCLC. These differences are mainly due to different transcriptional activities of the partner genes. Lastly, the regulation of TKF expression may depend on miRNAs/translational regulation and TKF degradation. Overall, these data provide empirical support to the concept of global neutrality, or near-neutrality (i.e. very weak selection of the mutant clone in the early phases of neoplastic transformation) for TKFs [47].

### Are TKFs required for the maintenance of a neoplastic phenotype?

It is generally believed that TKFs contribute to the maintenance of the neoplastic phenotype. This is well exemplified by the therapeutic success of TK inhibitors (TKi) for the treatment of chronic/indolent hematologic disorders (CML and CEL) [48, 49]. The oncogenic contribution of TKF to tumor maintenance can nevertheless vary according to several variables, including: (i) the properties of each fusion; (ii) the cellular context and the tumor microenvironment; (iii) the disease stage and evolution phase [28, 49]; (iv) the degree of oncogenic addiction to the TKF (e.g. ROS1-positive NSCLCs show better response to Crizotinib than ALK-positive NSLCLs) [50]; (v) the presence of concomitant genomic aberrations [30, 51]. In particular, the occurrence of multiple genetic defects may influence the response to therapy. This is the case of genetically complex disorders (e.g. ALL or end-stage solid cancers), which show a very aggressive clinical behavior and only partial responses to TKi [52]. The same holds true for some cases of ALK+ ALCL. The majority of ALK+ ALCL patients have a fairly good outcome with conventional chemotherapy treatments. However, rapid and even leukemic progression can occur. These are frequently associated with disrupting events, including *TP53* and *Blimp1* deletions [53] and *c*-*myc* translocation/amplifications. The acquisition of secondary events may indeed be linked to a more aggressive phenotype and can damper the addiction to ALK signaling [15]. This mechanism has also been reported in CML in blast crisis [54] and in ALK-positive NSLCLs resistant to Crizotinib. In the first case, BCR-ABL induces an IGF1 autocrine loop via Hck and Stat5b, in the latter case, the IGF-1R signaling synergizes with (and overcome) ALK by engaging the adaptor protein IRS-1 [51]. Moreover, restoration of p16 (INKa) or p14 (ARF) into Ph-positive leukemic cells (both CML in blast crisis and ALL) is able to determine cell growth arrest and/or apoptosis suggesting a pathogenic role for these deletions and their co-operating role with BCR-ABL [39].

### TKFs in hematopoietic disorders

Among hematologic malignancies, distinct subsets of acute leukemias, MPNs and non-Hodgkin lymphomas harbor specific TKFs (Table 1).

The most frequent TKFs involve *ABL-1* and *PDFGRa/b*, but translocations of *ARG*, *JAK2*, *NTRK3* and *SYK* have been reported, as well. Not all partner genes provide oligodimerization domains, as in the case of the FIP1L1-PDGFRa fusion, which leads to the activation of the kinase moiety by disruption of its juxtamembrane regulatory motif [55]. Alternatively, the partners do not interact directly, but engage intermediate proteins leading to an indirect dimerization (e.g. CTCCL-ALK, NUP214-ABL, FOP-FGFR1).

The t(9;22)(q34;q11) translocation juxtaposes the *BCR* and *ABL1* genes in virtually all cases of CML and in a subset of adult/pediatric ALL. The latter may represent a *de novo* disease or, as in many adults, the blastic transformation of CML. Of note, adult and (more frequently) pediatric ALL carry other TKFs, which are associated with a Ph-like phenotype [9]. The oncogenic properties of such fusions have been demonstrated in a large variety of *in vitro* and *in vivo* systems [9, 56, 57]. The second most common TKFs involve PDGFRα, PDGFRβ and FGFR1. Such TKFs characterize a group of hematological disorders, sharing similar clinico-pathological features, such as peripheral blood eosinophilia, clinical presentation as MPN and/or lymphoid tumors, and, at least for PDGFRα and PDGFRβ fusions, good response to Imatinib. For this reason, the 2008 WHO Classification of hematopietic and lymphoid tumors has introduced a separate diagnostic category for such disorders, referred to as “Myeloid and Lymphoid Neoplasms with Eosinophilia and Abnormalities of PDGFRα, PDGFRβ or FGFR1” [58].

Among mature T-cell neoplasms, subsets of Peripheral T-cell Lymphomas, not otherwise specified (PTCL, NOS) and Angioimmunoblastic T-cell Lymphomas (AITL) display *SYK* translocations and ITK-SYK fusion proteins [59-61]. ALK chimeras have been reported in a subset of ALCL (ALK-positive ALCL) [59], which are again recognized as a specific entity by the 2008 WHO Classification. Of note, the aberrant expression of ITK-SYK and NPM-ALK fusions in mice can lead to T-cell transformation [61, 62]. In particular, ITK-SYK mediates lymphomagenesis through the N-terminal phosphatidylinositol 3,4,5-trisphosphate-binding pleckstrin homology (PH) domain of ITK and SLP-76. ALK fusions, on the other hand, require the kinase domain and its flanking regions to activate multiple signaling pathways [59, 63]. Novel translocations involving *TYK2* and *ROS-1* in CD30-positive cutaneous lymphoproliferative disorders [64] and systemic ALK-negative ALCL [16] have been previously described. Interestingly, in addition to *NPM-1*, *NFkB2* and *NCOR2* are partners of these new TKFs and contribute to the neoplastic phenotype [16]. Finally, precursor T-cell neoplasms (i.e. T-cell acute lymphoblastic lymphoma [T-ALL]) may also harbor TKFs, which usually involve the *ABL* gene. Some of such translocations are also observed in B-ALL (e.g. ABL-NUP214), while others seem to be specific of T-ALL (ABL-EML1) [65] (Table 1). Interestingly, several lymphoproliferative disorders also display TK oncogenic mutations, further demonstrating the tumorigenic role of deregulated kinase signaling in hematopoietic disorders [66, 67].

TKFs are less frequent in mature non-Hodgkin B-cell neoplasms, with the notable exception of ALK-positive diffuse large B-cell lymphoma, in which the TKFs bear distinct oncogenic and clinico-pathological features [68] (Table 1).

### TKFs in solid tumors

In solid tumors, aberrant TK activity results from diverse mechanisms, including: (i) point mutations; (ii) gene amplifications; (iii) gene overexpression; and (iv) chromosomal translocations [51, 69, 70]. Abnormal TK activity can also result from *de novo* transcription initiation (ATI) sites, which lead to the synthesis of truncated intracellular TKDs (e.g. ALK^ATI^ in melanoma or ERBB4^ATI^ in ALCL) [71, 72].

In recent years, a wide array of recurrent translocations has been reported in solid tumors (Table 2) [14, 28]. In a systematic survey of nearly 7000 samples from the Cancer Genome Atlas, Stransky *et al.* have depicted an extremely variegated translocation landscape in solid tumors [5], which can display heterogeneous chromosomal translocations, with specific malignancies (such as sarcomas) being characterized by a high percentage of non-recurrent translocations and/or infrequent but recurrent gene fusions.

To date, fusions involving 14 different TKs have been described in solid tumors (Table 1). ALK is translocated in several tumors, including lung, colorectal, breast, renal, thyroid carcinomas and soft tissue tumors.

To date, fusions involving 14 different TKs have been described in solid tumors (Table 1). ALK is translocated in several tumors, including lung, colorectal, breast, renal, thyroid carcinomas and soft tissue tumors. Fusion partners include *EML4*, *TFG*, *KIF5B*, *KLC1* and *STRN* [28]. Rare translocations involving *SMEK2* (rectal adenocarcinoma) or *GTF*2IRD1 (thyroid carcinoma) have also been reported.

Next generation sequencing (NGS) analyses have highlighted new patterns of translocations, defining specific subgroups of common solid tumors (*BRAF* in melanoma; *FGFR1*, *FGFR2*, *FGFR3* in lung cancer; *NTRK* in thyroid cancer), and in clinically unmet patients (*BRAF* and *FGFR1* translocations in pediatric low-grade glioma; *NTRK1* and *NTRK2* in high-grade gliomas, and cholangiocarcinoma, Table 1). Lastly, MET is involved in chromosomal translocations of secondary glioblastomas and papillary renal carcinomas and *PI3KCA* translocations are observed in breast and prostate carcinomas.

The transforming activity of TKF in solid cancers has been extensively characterized through pre-clinical models [73], proving that classical MEK-ERK and PI3K-AKT pathways are critical players in sustaining the transformed phenotype and in mediating resistance to targeted therapies [74]. TKF dependency thus provides a molecular explanation for the “oncogene addiction” of different TKF-driven tumors [75].

Mechanistically, multiple causes of chromosomal translocations have been predicted: (i) exposure to toxic agents (probably the major initiating event); (ii) exposure to ionizing radiation (e.g. in papillary thyroid carcinoma); (iii) oxidative stress and chemotherapeutic agents (e.g. topoisomerase inhibitors).

## DISCOVERY AND DETECTION OF TK FUSIONS

The oncogenic role of TKFs has prompted huge efforts to improve their detection in cancer patients to better guide patient-directed treatment strategies [76].

Affinity purification coupled with mass spectrometry (AP-MS) is a powerful tool to characterize aberrant gene products and protein-protein interactions (PPIs). AP-MS has been successfully used to discover novel TKFs in NSCLC (ALK and ROS1) [77] and rare TKFs in other tumor histotypes [78]. AP-MS could also define activating mutations linked to TKi-resistant phenotypes [79]. Furthermore, proteomic patterns distinguish normal from pathological tissues and correctly classify primary/metastatic lesions, allowing tailored treatments and a better stratification of NSCLC [80].

The introduction of NGS-based approaches has rapidly enriched the overall knowledge of TKF, giving a better representation of their distribution in human cancers. In hematologic malignancies, NGS studies of Ph-like ALL have detected TKFs in >90% of patients [8]. Technically, both RNAseq and Whole Exome Sequence (WES) made these achievements possible. In some cases an exon capture specific for TK genes followed by massive parallel sequencing was applied [81-84]. Since these analyses can be performed on formalin-fixed paraffin-embedded samples, they can be applied to routine clinical practice. The possibility to test archived material will also allow for the characterization of the clonal evolution of TKF-bearing tumors, with consequent implementation of cancer tailored therapies.

### Clinical diagnostic tests: tumor fingerprints

Clinically, TKF in human hematological cancers can be detected by an array of techniques. Genomic/conventional cytogenetics (CG) or FISH are commonly used for the detection of BCR-ALB translocations. Negative samples (<5% of cases) frequently carry variant translocations or cryptic rearrangements, which can occasionally be detected by FISH. Both approaches are also used to monitor drug responses and disease evolution. FISH and RT-PCR have shown higher sensitivity at diagnosis and better MRD detection than CG. In particular, qRT-PCR has become the gold standard for the diagnosis of MRD [49]. FuseFISH probe-based approaches for the detection of fusion transcripts have also recently emerged [85], but their usefulness in clinical practice is uncertain.

Resistance to TKi treatments is currently detected by RT-PCR coupled with DNA Sanger sequencing, but new methods have been developed. Massive parallel sequencing is rapidly replacing the above reported strategies, as it can also identify subclonal populations and characterize tumor clonal heterogeneity [86]. Analogous approaches have now been extended to the detection of actionable mutations, copy number variations, and gene rearrangement in clinical cancer specimens.

In solid tumors, TKFs are clinically identified by several techniques, some of which are performed as companion tests to select the best TKi agent. In 2013, the USA and China's FDA approved FISH- and IHC-based diagnostic tests to identify *ALK* fusions or ALK protein expression in NSCLC. Similar strategies were proposed to assess *ROS1* and *RET* fusions [87]. Alternatively, a NGS-based detection kit for *RET*-rearrangements has been used to enroll patients in NSLCL clinical trials (Eisai sponsored trial; NCT01829217). It is conceivable that screening approaches to detect simultaneously multiple fusions will be implemented in the clinical arena [88].

Since different TKFs can be present in distinct cancer subsets, multi-targeted platforms have emerged as powerful tools to screen diagnostic samples. As multi-gene screening methods are entering into routine diagnostic procedures, new technical and therapeutic questions need to be addressed. Indeed, the greater efficacy of TKi compared to conventional treatments needs to be established for naïve patients carrying targetable fusions. Similarly, patients treated with multiple rounds of chemotherapeutic regimens need to be evaluated for the compliance to selective TKi, as limited data are available to justify their usage.

## TUMOR-ASSOCIATED TK TRANSLOCATIONS: CURRENT THERAPEUTIC STRATEGIES

The discovery of recurrent tumor-associated TK translocations has brought new strategies for the treatment of hematologic and non-hematologic tumors. In the last decades, TKi have revolutionized the treatment of CML, Ph-positive B-ALL [89] and NSCLC carrying *ALK* or *ROS-1* gene rearrangements [14, 90].

### TKi-based therapies for the treatment of hematological tumors

*BCR-ABL1* gene rearrangements are reported in all cases of CML and in about 30% of adult B-ALL. The fusion gene usually results from the t(9;22)(q34;q11.2) translocation, but 5-10% of cases bear variants and/or cryptic translocations [2]. BCR-ABL1 oncogenic properties are due to the constitutive activation of ABL1 TK [91, 92] and the loss of ABL-1 N-terminus regulatory domain.

Clinically, Imatinib has revolutionized the prognosis and outcome of CML, converting a highly lethal hematological neoplasm to a chronic and curable disease [49]. Complete cytogenetic responses (CCyR) are indeed reported in 87% of Imatinib-treated patients, with a progression-free survival (PFS) of 93% [93]. After 2 years of sustained CCyR, the life expectancy for CML patients is comparable to that of the general population [94]. Despite these results, several studies pinpointed a subset of cases with primary/secondary resistance to Imatinib, which can be overcome by new generation TKi (i.e. Nilotinib, Dasatinib and Bosutinib) with improved clinical efficacy [95]. These lines of evidence led the European Leukemia Net to recommend Imatinib, Nilotinib and/or Dasatinib as first line drugs for the treatment of CML. More aggressive therapeutic approaches (e.g. hematopoietic stem cell transplant) are considered only in very selected cases (e.g. refractory/relapsing cases unresponsive to TKi or accelerated/blast phase CML) [49].

In CML patients, resistance to TKi can result from two major mechanisms: (i) occurrence of TKi-resistant BCR-ABL1 mutations; (ii) enhancement of BCR-ABL1-independent pro-survival pathways. BCR-ABL1-dependent resistance has been overcome by third generation TKi that also target the most common ABL1 mutations (e.g. BCR-ABL1^T315I^). The most promising therapeutic agents are the conventional TKi, Ponatinib, and the BCR-ABL1 switch control inhibitor, DCC-2036 [96]. Notably, good results with Ponatinib have been obtained even in accelerated and blast phase CML [97], while the distinct action of DCC-2036 may overcome pre-existing mutations, including T315I [96]. Pemovska *et al.* have recently re-proposed the utility of Axitinib, an anti-VEGFR molecule approved for renal cell carcinoma, to counteract the resistance phenotype of T315I gatekeeper CML. Indeed, Axitinib tightly binds the T315I mutated kinase and inhibits leukemic cell growth, showing a better toxicity profile than other new-generation drugs, such as Ponatinib [98].

BCR-ABL1-independent resistance is mainly due to the activation of alternative signaling pathways promoting neoplastic cell survival [99]. Such pathways can be either extrinsic (i.e. activated by the marrow microenvironment) or intrinsic (i.e. due to microenvironment-independent mechanisms). Both intrinsic and extrinsic pathways lead to the activation of STAT3, through its phosphorylation at Tyr-705 [100]. Combined inhibition of STAT via BP-5-087, a potent and selective STAT3 SH2 domain inhibitor and BCR-ABL1 leads to synthetic lethality in resistant CML and constitutes a very promising therapeutic approach for refractory/relapsing CML [100]. Another therapeutic opportunity is represented by glitazones, anti-diabetic drugs that down-regulate STAT5 expression by inhibiting the peroxisome proliferator-activated receptor-γ (PPARγ). Recent data show that their use may lead to the complete eradication of leukemic stem pools, thus greatly improving the survival of CML patients in sustained CMR [101].

Unlike what has been observed in CML, the use of single-agent TKi in Ph-positive ALL has not produced sustained clinical responses. This is probably due to the rapid development of TK mutations, which are reported in >80% of adult Ph-positive ALLs at relapse [102]. Ph-positive ALL and CML also greatly differ in terms of genomic backgrounds, which may influence the response to TKi. For instance, INK4-ARF deletions, which often occur in acute Ph-positive ALL with aggressive clinical course, facilitate the emergence of BCR-ABL drug-resistant clones. This molecular event is not observed in indolent CML or in CML in blast crisis [103].

These data provide the rationale for the use of multi-modal regimens for the treatment of Ph-positive ALL, based on a combination of intensive chemotherapy and TKi. Very encouraging results have been first obtained in pediatric trials (COG, EsPhALL and SHOP-2005 trials) [104, 105] and, more recently, in adult patients [106]. In particular, a recent Phase II trial on Imatinib mesylate *plus* HyperCVAD (Cyclophosphamide, Vincristine, Doxorubicine, Dexamethasone) has reported complete clinical remission in 93% of adults with active Ph-positive ALL, with a 5-year overall survival (OS) of 43%. Notably, allogeneic stem cell transplant (ASCT) after Imatinib and HyperCVAD improved median OS only in patients with residual molecular disease. The role of Dasatinib and Nilotinib for the treatment of pediatric and adult Ph-positive ALL is still under evaluation, but preliminary results indicate their safety and efficacy when used with high dose chemotherapy and/or ASCT [107]. Encouraging data have also been obtained with Dasatinib and corticosteroid, which proves the potential efficacy of chemo-free protocols [108]. Finally, the association of TKi and bispecific T-cell engaging antibodies (BiTE) or chimeric antigen receptors T-cells (CARTs) represents a promising strategy for the treatment of Ph-positive ALL [109].

### TKI-based therapies for the treatment of solid tumors

The introduction of TKi for the treatment of ALK-, ROS1- and RET-translocated NSCLC [14, 110, 111] has led to an astonishing improvement of patients' outcome and survival. More recently, fusions involving *NTRK1* and *FGFR1/2/3* have been reported (Table 1), thus enlarging the spectrum of promising therapies for NSCLC (i.e. Crizotinib, Ceritinib, Alecitinib for ALK-positive NSCLC; Crizotinib for ROS1-positive NSCLC; Cabozantinib and Vandetanib for RET-positive NSCLC).

Crizotinib represents the paradigm of efficient translation of molecular knowledge to the bedside. Initially developed as a c-Met inhibitor, Crizotinib showed clinical activity on advanced ALK-positive NSCLC in both the PROFILE 1001 (phase I) and PROFILE 1014 (phase III) clinical trials. Of note, in such tumors, Crizotinib displayed even greater efficacy than standard chemotherapy [112]. Similar results have been observed in ROS1- or RET-positive NSCLCs, with good responses to Crizotinib in ROS1-positive cases [14] and to Cabozantinib in Crizotinib-refractory cells [113]. The application of TKi has recently expanded to RET-mutated medullary thyroid carcinoma, RET-translocated NSCLC and other solid tumors (NCT02183870, NCT0194502, NCT01823068, NCT01639508). More recently, inhibitors with broad spectrum of activities were tested (RXDX-101), providing novel strategies to target different molecular defects (NTRK1/2/3, ROS1 and ALK translocation) shared by wide range of solid tumors (NCT02568267, NCT02097810).

Despite these outstanding results, TKi often lose their efficacy due to the development of treatment-related resistance [114]. Much like hematologic neoplasms, primary and secondary mechanisms determine resistance to TKi in solid tumors. Primary resistance is linked to the tridimensional structures of the protein that confer TKi-resistance [115], whereas secondary resistance mainly consists of gene mutations, bypass mechanisms, and/or gene amplifications overcoming the effect of TKi at therapeutic levels.

As for target mutations, different genetic alterations associated with resistance have been described for ALK-positive NSCLC: i) L1169M within the “gatekeeper” domain (the most frequent); ii) L1152R, associated with EGFR signaling activation; iii) G1202R and S1206Y within the ATP-binding pocket, leading to a decreased Crizotinib affinity [116]. The most frequently reported bypass mechanisms associated with secondary resistance to ALK inhibition are: i) EGFR/KRAS mutations [117] and Tyrosine Kinase Receptor (ERBB, c-MET, etc.) activation triggered by paracrine stimuli [118, 119]; ii) c-KIT amplification, requiring SCF [120], IGF-1R up-regulation [51] and recruitment of P2Y receptors [121]. Other resistance mechanisms include impaired drug influx (OCT-1), increased drug efflux (MDR1), or microenvironment-mediated resistance [122].

Different strategies have been proposed to overcome TKi acquired resistance, including the development of next-generation drugs, such as the ALK-inhibitors LDK378-Ceritinib and AP26113-Brigatinib (for ALK-positive NSCLC resistant to Crizotinib), Lecitinib (for ALK-positive NSCLC carrying the L1169M mutation) and PF06463922 (a dual ALK/ROS1 inhibitor with a good CNS penetration profile) [123]. Additionally, Hsp-90 inhibitors (AUY922-Ganetespib) are currently under evaluation in phase I/II clinical trials. Other emerging strategies include the administration of combined therapies to by-pass the “addiction” to single TKFs (e.g., combined inhibition of HER family [Dacomitinib], c-KIT [Dasatinib], src [Saracatinib], MEK [AZD6244], and IGF-1R [Linsitinib]). The re-timing of drug administration should also be considered, as recently demonstrated by the case of a Crizotinib-resistant ALK-positive NSCLC, displaying good response to this TKi after a withdrawal period [124]. Similar results have been obtained in an EGFR-mutated NSCLC, which regained sensitivity to EGFR-inhibitors upon therapy discontinuation [125]. Lastly, immunotherapeutic strategies (i.e. vaccination against over-expressed oncogenic antigens) may represent an effective approach for solid tumors, as recently reported in ALK-positive disorders [59, 126].

A final major roadblock concerning TKi is the poor response of central nervous system (CNS) metastases to therapy. This drawback may be overcome by different strategies: (i) combined therapies with drugs favoring TKi CNS accumulation, as recently attempted through the co-administration of Vandetanib and Everolimus (an immunosuppressive drug also modulating the P-glycoprotein efflux activity) [127]; and (ii) usage of single compound with higher brain penetrance, such as Ceritinib compared to Crizotinib.

## FUTURE PERSPECTIVES

**Figure 2:**
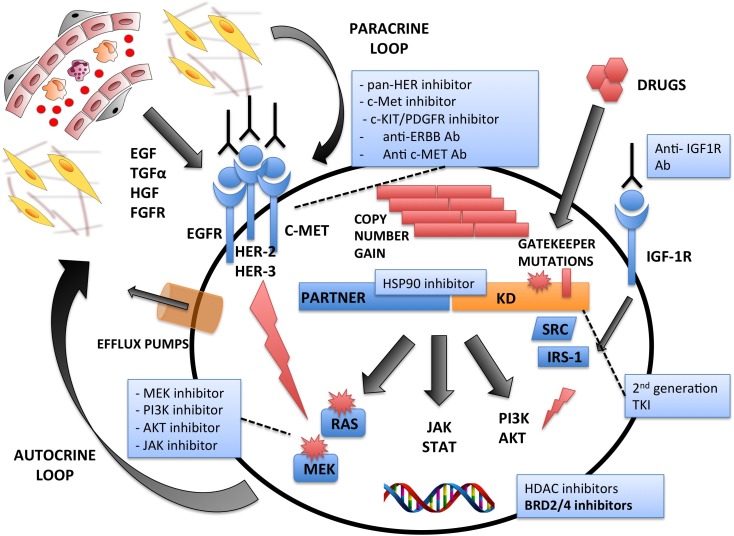
Mechanisms of Intrinsic and/or Acquired Resistances to Tyrosine Kinase Exposure Multiple mechanisms are associated with the emergence of drug resistance. These include: development of secondary mutations in the Kinase Domain (KD) at gatekeeper sites; copy number gains; activation of alternative oncogenic pathways via somatic mutations (i.e. RAS), and compensatory “by-pass” routes by Receptor Tyrosine Kinases signaling (i.e. EGFR, HER-2, HER-3, c-MET). Alternatively, resistance may also be due to either paracrine external signals through the tumor microenvironment, or via autocrine loops. The major drug categories and their therapeutic modalities are indicated.

Despite TKi have dramatically changed the clinical management of numerous cancers, many questions have still to be answered. One of the major problems concerning TKi is the high frequency of primary refractoriness/tumor relapses [128] (Figure 2). Possible solutions for this limitation are: (i) the development of more potent drugs with limited side effects; (ii) the co-administration of TKi and chemotherapeutic agents [129], immune-based protocols [130] and/or bone marrow transplant [131]; and (iii) further research on treatment timing and dosage.

Of note, a growing number of protocols combining chemotherapy and TKi are currently being tested in both TKF-induced hematological and solid tumors [107, 132] [133], and several trials are evaluating the efficacy of new TKi either alone or in combination with more conventional therapies (NCT02269085; NCT02427620; NCT02315768; NCT01606878; NCT02134912; NCT02511184). Preclinical studies have also demonstrated a substantial advantage of combining classical chemotherapy with TKi: Das and colleagues showed a synergistic effect of Crizotinib and Temozolomide in FIG-ROS1-positive glioblastoma patient-derived cells, paving a route to improve clinical outcome [134]. Similarly, Appelmann and colleagues evaluated the possibility of combining Dasatinib, Ruxolitinib and Dexamethasone to prolong remission and avoid major toxicities in a mouse model of Ph-positive ALL [135].

One of the most challenging issues on the combined use of TKi and chemotherapeutics concerns the optimal doses and schedules to use. Toward this end, mathematical models might improve the clinical success, limiting unacceptable side effects and TKi/conventional therapy-resistance [136]. This approach also needs dedicated trials, the cooperative agreement of pharmaceutical companies and new regulations on intellectual and commercial issues. Moreover, we need to develop biological and mathematical models to predict cancer evolution, capable of predicting cancer evolutionary trajectories based on pre-treatment diversity. Lastly, we foresee that these predictions need to be adjusted in each individual patient by integrating longitudinally acquired molecular readouts representative of the global cancer landscape (i.e. comprehensive genomic data [WES] of multiple sites, liquid biopsies, etc.).

Other questions regarding TKi concern the risks and benefits of second/third generation drugs. Thus, the use of first generation compounds remain a valuable option in many settings, including highly targeted diseases and the management of third world populations. The increasing health costs, the uncontrolled fee schedules in many industrialized countries, the increasing co-payer costs and the accessibility of generic TKi represent very tangible issues which may be solved only via international based supports and regulations. Finally, the need of cutting health system costs poses unprecedented challenges to molecular medicine programs. In fact, the success of such therapies depends on the characterization of individual cancers (both primary and recurrent/metastatic lesions). These analyses have to be performed by a pool of medical centers following certified programs, with consequent huge health costs.

**Figure 3:**
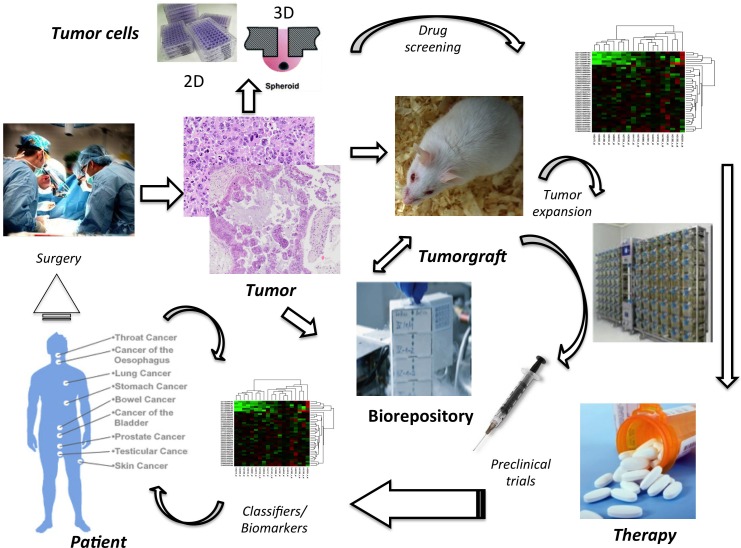
Comprehensive Management of Tyrosine Kinase-driven Cancers Molecular and functional characterization of human cancers in combination with drug screening tests on primary patient tumor samples is expected to drive precise target therapies. It is anticipated that tumor samples will be used to generated 2D/3D and well as PDTX models. *In vitro* models from primary samples or from PDTX will serve to screen large libraries of compounds, whose efficacy will be established using a battery of biological and molecular readouts. The data emerging from these high throughput screenings will be pivotal to test selected molecules in preclinical PDTX-based trials. Extensive molecular and functional readouts will then be obtained from both tumors and host compartments (i.e. plasma/liquid biopsy) to create a detailed profile of *in vivo* classifiers and biomarkers. These will ultimately serve as surrogates to predict and define response to specific therapies in cancer patients. The development of tumor biorepositories of primary and metastatic lesions, cell lines and PDTX from primary human cancers is predicted to lead to the recognition and understanding of new oncogenic events. It is anticipated that effective targeted therapies will improve clinical responses and ultimately will lead to lower health care costs.

These practical problems are paralleled by a series of scientific and methodological challenges. Although the genomic characterization of each cancer has originally prompted the use of selective compounds, it is now clear that this approach may not be sufficient. Indeed, favorable responses to selective drugs are not strictly linked to defined genetic defects. Thus, functional tests (e.g. comprehensive phosphorylation maps) should be performed together with genomic analyses. As an alternative, *in vitro* preclinical tests on patient-derived cell lines or PDTX may be introduced (Figure 3).

Of note, *in vitro* tests with patient-derived tissue samples may allow personalized pharmacologic interventions without the limits related to the use of immortalized cell lines or other tumor models. Patient-derived cancer models are indeed pivotal for drug discovery, for the characterization of cancer biology and for the assessment of personalized therapies [137-139]. Several cancer PDTX models have been developed, but only handful of them carries TKFs [15, 140-142]; thus, a more systematic effort in generating “ad hoc” models is highly recommended.

We anticipate that *in vitro* primary cell lines will allow for the rapid testing of drug libraries, with subsequent validation in more complex models (e.g. PDTX). It is also desirable that new clinical trials will include parallel co-clinical mouse PDTX-trials, which will establish the efficacy of such models. The molecular stratification of these models will finally provide new cancer classifiers with possible diagnostic/prognostic implications (Figure 3).

The considerable therapeutic progress against TKF-driven tumors may also prove beneficial for the treatment of those translocation-negative neoplasms, which nonetheless carry defects of TKF-related signaling pathways. As previously highlighted, TKFs indeed represent only one of the several mechanisms leading to TK dysfunction, and other TK impairments (i.e. gene mutations/amplifications or non-fusion rearrangements) lead to phenotypes closely resembling those of TKF-driven neoplasms. This is, for instance, the case of subsets of ALK-negative ALCL, carrying JAK1 and STAT3 activating mutations, which induce transcriptional signatures akin to those of ALK-positive ALCL [16]. Moreover, it is now recognized that activating mutations can synergize with TFKs and lead to full neoplastic transformation [8]. This molecular evidence constitutes the rationale for the administration of TKi compounds in specific subsets of both TKF-negative and TFK-positive tumors. Further studies and clinical trials will help clarify this captivating opportunity.

In conclusion, it is desirable that the academic community promotes dedicated programs on TKi and TKF-bearing cancers, also encouraging the support of pharmaceutical companies and the distribution of compounds under compassionate use. This approach, together with the creation of open-access clinical databases, will greatly contribute to the understanding of cancer-related TKFs and to a better management of (TKF-positive and TKF-negative) cancer patients.

## References

[1] Nowell PC (1960). A minute chromosome in human chronic granulocitic leukemia. Science.

[2] Rowley JD (1973). Letter: A new consistent chromosomal abnormality in chronic myelogenous leukaemia identified by quinacrine fluorescence and Giemsa staining. Nature.

[3] Heisterkamp N, Stam K, Groffen J, de Klein A, Grosveld G (1985). Structural organization of the bcr gene and its role in the Ph' translocation. Nature.

[4] Heisterkamp N, Stephenson JR, Groffen J, Hansen PF, de Klein A, Bartram CR, Grosveld G (1983). Localization of the c-ab1 oncogene adjacent to a translocation break point in chronic myelocytic leukaemia. Nature.

[5] Stransky N, Cerami E, Schalm S, Kim JL, Lengauer C (2014). The landscape of kinase fusions in cancer. Nat Commun.

[6] Mullighan CG, Collins-Underwood JR, Phillips LA, Loudin MG, Liu W, Zhang J, Ma J, Coustan-Smith E, Harvey RC, Willman CL, Mikhail FM, Meyer J, Carroll AJ, Williams RT, Cheng J, Heerema NA (2009). Rearrangement of CRLF2 in B-progenitor- and Down syndrome-associated acute lymphoblastic leukemia. Nature genetics.

[7] Hantschel O (2012). Structure, regulation, signaling, and targeting of abl kinases in cancer. Genes & cancer.

[8] Roberts KG, Li Y, Payne-Turner D, Harvey RC, Yang YL, Pei D, McCastlain K, Ding L, Lu C, Song G, Ma J, Becksfort J, Rusch M, Chen SC, Easton J, Cheng J (2014). Targetable kinase-activating lesions in Ph-like acute lymphoblastic leukemia. N Engl J Med.

[9] Roberts KG, Morin RD, Zhang J, Hirst M, Zhao Y, Su X, Chen SC, Payne-Turner D, Churchman ML, Harvey RC, Chen X, Kasap C, Yan C, Becksfort J, Finney RP, Teachey DT (2012). Genetic alterations activating kinase and cytokine receptor signaling in high-risk acute lymphoblastic leukemia. Cancer Cell.

[10] Valent P (2014). Targeting the JAK2-STAT5 pathway in CML. Blood.

[11] Medves S, Demoulin JB (2012). Tyrosine kinase gene fusions in cancer: translating mechanisms into targeted therapies. J Cell Mol Med.

[12] Tan L, Wang J, Tanizaki J, Huang Z, Aref AR, Rusan M, Zhu SJ, Zhang Y, Ercan D, Liao RG, Capelletti M, Zhou W, Hur W, Kim N, Sim T, Gaudet S (2014). Development of covalent inhibitors that can overcome resistance to first-generation FGFR kinase inhibitors. Proc Natl Acad Sci U S A.

[13] Cowan-Jacob SW, Mobitz H, Fabbro D (2009). Structural biology contributions to tyrosine kinase drug discovery. Curr Opin Cell Biol.

[14] Shaw AT, Engelman JA (2014). Ceritinib in ALK-rearranged non-small-cell lung cancer. N Engl J Med.

[15] Abate F, Todaro M, van der Krogt JA, Boi M, Landra I, Machiorlatti R, Tabbo F, Messana K, Abele C, Barreca A, Novero D, Gaudiano M, Aliberti S, Di Giacomo F, Tousseyn T, Lasorsa E (2015). A novel patient-derived tumorgraft model with TRAF1-ALK anaplastic large-cell lymphoma translocation. Leukemia.

[16] Crescenzo R, Abate F, Lasorsa E, Tabbo F, Gaudiano M, Chiesa N, Di Giacomo F, Spaccarotella E, Barbarossa L, Ercole E, Todaro M, Boi M, Acquaviva A, Ficarra E, Novero D, Rinaldi A (2015). Convergent Mutations and Kinase Fusions Lead to Oncogenic STAT3 Activation in Anaplastic Large Cell Lymphoma. Cancer Cell.

[17] Bonvini P, Gastaldi T, Falini B, Rosolen A (2002). Nucleophosmin-anaplastic lymphoma kinase (NPM-ALK), a novel Hsp90-client tyrosine kinase: down-regulation of NPM-ALK expression and tyrosine phosphorylation in ALK(+) CD30(+) lymphoma cells by the Hsp90 antagonist 17-allylamino,17-demethoxygeldanamycin. Cancer Res.

[18] Yao Q, Nishiuchi R, Li Q, Kumar AR, Hudson WA, Kersey JH (2003). FLT3 expressing leukemias are selectively sensitive to inhibitors of the molecular chaperone heat shock protein 90 through destabilization of signal transduction-associated kinases. Clinical cancer research.

[19] De Keersmaecker K, Rocnik JL, Bernad R, Lee BH, Leeman D, Gielen O, Verachtert H, Folens C, Munck S, Marynen P, Fornerod M, Gilliland DG, Cools J (2008). Kinase activation and transformation by NUP214-ABL1 is dependent on the context of the nuclear pore. Mol Cell.

[20] Armstrong F, Lamant L, Hieblot C, Delsol G, Touriol C (2007). TPM3-ALK expression induces changes in cytoskeleton organisation and confers higher metastatic capacities than other ALK fusion proteins. Eur J Cancer.

[21] Grieco M, Santoro M, Berlingieri MT, Melillo RM, Donghi R, Bongarzone I, Pierotti MA, Della Porta G, Fusco A, Vecchio G (1990). PTC is a novel rearranged form of the ret proto-oncogene and is frequently detected *in vivo* in human thyroid papillary carcinomas. Cell.

[22] Greco A, Pierotti MA, Bongarzone I, Pagliardini S, Lanzi C, Della Porta G (1992). TRK-T1 is a novel oncogene formed by the fusion of TPR and TRK genes in human papillary thyroid carcinomas. Oncogene.

[23] Butti MG, Bongarzone I, Ferraresi G, Mondellini P, Borrello MG, Pierotti MA (1995). A sequence analysis of the genomic regions involved in the rearrangements between TPM3 and NTRK1 genes producing TRK oncogenes in papillary thyroid carcinomas. Genomics.

[24] Tognon C, Knezevich SR, Huntsman D, Roskelley CD, Melnyk N, Mathers JA, Becker L, Carneiro F, MacPherson N, Horsman D, Poremba C, Sorensen PH (2002). Expression of the ETV6-NTRK3 gene fusion as a primary event in human secretory breast carcinoma. Cancer Cell.

[25] Knezevich SR, McFadden DE, Tao W, Lim JF, Sorensen PH (1998). A novel ETV6-NTRK3 gene fusion in congenital fibrosarcoma. Nature genetics.

[26] Blume-Jensen P, Hunter T (2001). Oncogenic kinase signalling. Nature.

[27] Krause DS, Van Etten RA (2005). Tyrosine kinases as targets for cancer therapy. N Engl J Med.

[28] Shaw AT, Hsu PP, Awad MM, Engelman JA (2013). Tyrosine kinase gene rearrangements in epithelial malignancies. Nature reviews Cancer.

[29] Buitenhuis M, Verhagen LP, Cools J, Coffer PJ (2007). Molecular mechanisms underlying FIP1L1-PDGFRA-mediated myeloproliferation. Cancer Res.

[30] Yamada Y, Sanchez-Aguilera A, Brandt EB, McBride M, Al-Moamen NJ, Finkelman FD, Williams DA, Cancelas JA, Rothenberg ME (2008). FIP1L1/PDGFRalpha synergizes with SCF to induce systemic mastocytosis in a murine model of chronic eosinophilic leukemia/hypereosinophilic syndrome. Blood.

[31] Daley GQ, Van Etten RA, Baltimore D (1990). Induction of chronic myelogenous leukemia in mice by the P210bcr/abl gene of the Philadelphia chromosome. Science.

[32] Elefanty AG, Hariharan IK, Cory S (1990). bcr-abl, the hallmark of chronic myeloid leukaemia in man, induces multiple haemopoietic neoplasms in mice. EMBO J.

[33] Van Etten RA (2001). Models of chronic myeloid leukemia. Curr Oncol Rep.

[34] Foley SB, Hildenbrand ZL, Soyombo AA, Magee JA, Wu Y, Oravecz-Wilson KI, Ross TS (2013). Expression of BCR/ABL p210 from a knockin allele enhances bone marrow engraftment without inducing neoplasia. Cell Rep.

[35] Ismail SI, Naffa RG, Yousef AM, Ghanim MT (2014). Incidence of bcrabl fusion transcripts in healthy individuals. Mol Med Rep.

[36] Mahon FX, Rea D, Guilhot J, Guilhot F, Huguet F, Nicolini F, Legros L, Charbonnier A, Guerci A, Varet B, Etienne G, Reiffers J, Rousselot P, Intergroupe Francais des Leucemies Myeloides C (2010). Discontinuation of imatinib in patients with chronic myeloid leukaemia who have maintained complete molecular remission for at least 2 years: the prospective, multicentre Stop Imatinib (STIM) trial. Lancet Oncol.

[37] Williams RT, Sherr CJ (2007). The ARF tumor suppressor in acute leukemias: insights from mouse models of Bcr-Abl-induced acute lymphoblastic leukemia. Adv Exp Med Biol.

[38] Iacobucci I, Ferrari A, Lonetti A, Papayannidis C, Paoloni F, Trino S, Storlazzi CT, Ottaviani E, Cattina F, Impera L, Abbenante MC, Vignetti M, Vitale A, Potenza L, Paolini S, Soverini S (2011). CDKN2A/B alterations impair prognosis in adult BCR-ABL1-positive acute lymphoblastic leukemia patients. Clinical cancer research.

[39] Bai Y, Lu Z, Lin Y, Sun B, Wang S, Wang G (2013). Restoration of INK4a/ARF gene inhibits cell growth and cooperates with imatinib mesylate in Philadelphia chromosome-positive leukemias. Oncology research.

[40] Hurtz C, Hatzi K, Cerchietti L, Braig M, Park E, Kim YM, Herzog S, Ramezani-Rad P, Jumaa H, Muller MC, Hofmann WK, Hochhaus A, Ye BH, Agarwal A, Druker BJ, Shah NP (2011). BCL6-mediated repression of p53 is critical for leukemia stem cell survival in chronic myeloid leukemia. J Exp Med.

[41] Lee TY, Ezelle HJ, Venkataraman T, Lapidus RG, Scheibner KA, Hassel BA (2013). Regulation of human RNase-L by the miR-29 family reveals a novel oncogenic role in chronic myelogenous leukemia. J Interferon Cytokine Res.

[42] Albers C, Illert AL, Miething C, Leischner H, Thiede M, Peschel C, Duyster J (2011). An RNAi-based system for loss-of-function analysis identifies Raf1 as a crucial mediator of BCR-ABL-driven leukemogenesis. Blood.

[43] Churchman ML, Low J, Qu C, Paietta EM, Kasper LH, Chang Y, Payne-Turner D, Althoff MJ, Song G, Chen SC, Ma J, Rusch M, McGoldrick D, Edmonson M, Gupta P, Wang YD (2015). Efficacy of Retinoids in IKZF1-Mutated BCR-ABL1 Acute Lymphoblastic Leukemia. Cancer Cell.

[44] Zhang Q, Wei F, Wang HY, Liu X, Roy D, Xiong QB, Jiang S, Medvec A, Danet-Desnoyers G, Watt C, Tomczak E, Kalos M, Riley JL, Wasik MA (2013). The potent oncogene NPM-ALK mediates malignant transformation of normal human CD4(+) T lymphocytes. Am J Pathol.

[45] Maes B, Vanhentenrijk V, Wlodarska I, Cools J, Peeters B, Marynen P, de Wolf-Peeters C (2001). The NPM-ALK and the ATIC-ALK fusion genes can be detected in non-neoplastic cells. Am J Pathol.

[46] Boland JM, Jang JS, Li J, Lee AM, Wampfler JA, Erickson-Johnson MR, Soares I, Yang P, Jen J, Oliveira AM, Yi ES (2013). MET and EGFR mutations identified in ALK-rearranged pulmonary adenocarcinoma: molecular analysis of 25 ALK-positive cases. J Thorac Oncol.

[47] Jankovic GM, Pavlovic M, Vukomanovic DJ, Colovic MD, Lazarevic V (2001). The fundamental prevalence of chronic myeloid leukemia-generating clonogenic cells in the light of the neutrality theory of evolution. Blood Cells Mol Dis.

[48] Baccarani M, Cilloni D, Rondoni M, Ottaviani E, Messa F, Merante S, Tiribelli M, Buccisano F, Testoni N, Gottardi E, de Vivo A, Giugliano E, Iacobucci I, Paolini S, Soverini S, Rosti G (2007). The efficacy of imatinib mesylate in patients with FIP1L1-PDGFRalpha-positive hypereosinophilic syndrome. Results of a multicenter prospective study. Haematologica.

[49] Baccarani M, Deininger MW, Rosti G, Hochhaus A, Soverini S, Apperley JF, Cervantes F, Clark RE, Cortes JE, Guilhot F, Hjorth-Hansen H, Hughes TP, Kantarjian HM, Kim DW, Larson RA, Lipton JH (2013). European LeukemiaNet recommendations for the management of chronic myeloid leukemia: 2013. Blood.

[50] Camidge DR, Bang YJ, Kwak EL, Iafrate AJ, Varella-Garcia M, Fox SB, Riely GJ, Solomon B, Ou SH, Kim DW, Salgia R, Fidias P, Engelman JA, Gandhi L, Janne PA, Costa DB (2012). Activity and safety of crizotinib in patients with ALK-positive non-small-cell lung cancer: updated results from a phase 1 study. Lancet Oncol.

[51] Lovly CM, McDonald NT, Chen H, Ortiz-Cuaran S, Heukamp LC, Yan Y, Florin A, Ozretic L, Lim D, Wang L, Chen Z, Chen X, Lu P, Paik PK, Shen R, Jin H (2014). Rationale for co-targeting IGF-1R and ALK in ALK fusion-positive lung cancer. Nature medicine.

[52] Ottmann OG, Wassmann B (2005). Treatment of Philadelphia chromosome-positive acute lymphoblastic leukemia. Hematology / the Education Program of the American Society of Hematology American Society of Hematology Education Program.

[53] Boi M, Rinaldi A, Kwee I, Bonetti P, Todaro M, Tabbo F, Piva R, Rancoita PM, Matolcsy A, Timar B, Tousseyn T, Rodriguez-Pinilla SM, Piris MA, Bea S, Campo E, Bhagat G (2013). PRDM1/BLIMP1 is commonly inactivated in anaplastic large T-cell lymphoma. Blood.

[54] Lakshmikuttyamma A, Pastural E, Takahashi N, Sawada K, Sheridan DP, DeCoteau JF, Geyer CR (2008). Bcr-Abl induces autocrine IGF-1 signaling. Oncogene.

[55] Stover EH, Chen J, Folens C, Lee BH, Mentens N, Marynen P, Williams IR, Gilliland DG, Cools J (2006). Activation of FIP1L1-PDGFRalpha requires disruption of the juxtamembrane domain of PDGFRalpha and is FIP1L1-independent. Proc Natl Acad Sci U S A.

[56] Toffalini F, Demoulin JB (2010). New insights into the mechanisms of hematopoietic cell transformation by activated receptor tyrosine kinases. Blood.

[57] Van Etten RA (2007). Oncogenic signaling: new insights and controversies from chronic myeloid leukemia. J Exp Med.

[58] Gotlib J (2015). World Health Organization-defined eosinophilic disorders: 2015 update on diagnosis, risk stratification, and management. American journal of hematology.

[59] Chiarle R, Voena C, Ambrogio C, Piva R, Inghirami G (2008). The anaplastic lymphoma kinase in the pathogenesis of cancer. Nature reviews Cancer.

[60] Streubel B, Vinatzer U, Willheim M, Raderer M, Chott A (2006). Novel t(5;9)(q33;q22) fuses ITK to SYK in unspecified peripheral T-cell lymphoma. Leukemia.

[61] Pechloff K, Holch J, Ferch U, Schweneker M, Brunner K, Kremer M, Sparwasser T, Quintanilla-Martinez L, Zimber-Strobl U, Streubel B, Gewies A, Peschel C, Ruland J (2010). The fusion kinase ITK-SYK mimics a T cell receptor signal and drives oncogenesis in conditional mouse models of peripheral T cell lymphoma. J Exp Med.

[62] Chiarle R, Gong JZ, Guasparri I, Pesci A, Cai J, Liu J, Simmons WJ, Dhall G, Howes J, Piva R, Inghirami G (2003). NPM-ALK transgenic mice spontaneously develop T-cell lymphomas and plasma cell tumors. Blood.

[63] Zamo A, Chiarle R, Piva R, Howes J, Fan Y, Chilosi M, Levy DE, Inghirami G (2002). Anaplastic lymphoma kinase (ALK) activates Stat3 and protects hematopoietic cells from cell death. Oncogene.

[64] Velusamy T, Kiel MJ, Sahasrabuddhe AA, Rolland D, Dixon CA, Bailey NG, Betz BL, Brown NA, Hristov AC, Wilcox RA, Miranda RN, Medeiros LJ, Jeon YK, Inamdar KV, Lim MS, Elenitoba-Johnson KS (2014). A novel recurrent NPM1-TYK2 gene fusion in cutaneous CD30-positive lymphoproliferative disorders. Blood.

[65] Hagemeijer A, Graux C (2010). ABL1 rearrangements in T-cell acute lymphoblastic leukemia. Genes, chromosomes & cancer.

[66] Tefferi A, Elliott M, Pardanani A (2015). Chronic neutrophilic leukemia: novel mutations and their impact on clinical practice. Current opinion in hematology.

[67] Quintas-Cardama A, Verstovsek S (2013). Molecular pathways: Jak/STAT pathway: mutations, inhibitors, and resistance. Clinical cancer research.

[68] Bubala H, Maldyk J, Wlodarska I, Sonta-Jakimczyk D, Szczepanski T (2006). ALK-positive diffuse large B-cell lymphoma. Pediatr Blood Cancer.

[69] Mizuki M, Ueda S, Matsumura I, Ishiko J, Schwable J, Serve H, Kanakura Y (2003). Oncogenic receptor tyrosine kinase in leukemia. Cell Mol Biol (Noisy-le-grand).

[70] Zandi R, Larsen AB, Andersen P, Stockhausen MT, Poulsen HS (2007). Mechanisms for oncogenic activation of the epidermal growth factor receptor. Cell Signal.

[71] Wiesner T, Lee W, Obenauf AC, Ran L, Murali R, Zhang QF, Wong EW, Hu W, Scott SN, Shah RH, Landa I, Button J, Lailler N, Sboner A, Gao D, Murphy DA (2015). Alternative transcription initiation leads to expression of a novel ALK isoform in cancer. Nature.

[72] Scarfo I, Pellegrino E, Mereu E, Kwee I, Agnelli L, Bergaggio E, Garaffo G, Vitale N, Caputo M, Machiorlatti R, Circosta P, Abate F, Barreca A, Novero D, Mathew S, Rinaldi A (2015). Identification of a new subclass of ALK negative ALCL expressing aberrant levels of ERBB4 transcripts. Blood.

[73] Soda M, Choi YL, Enomoto M, Takada S, Yamashita Y, Ishikawa S, Fujiwara S, Watanabe H, Kurashina K, Hatanaka H, Bando M, Ohno S, Ishikawa Y, Aburatani H, Niki T, Sohara Y (2007). Identification of the transforming EML4-ALK fusion gene in non-small-cell lung cancer. Nature.

[74] Hrustanovic G, Olivas V, Pazarentzos E, Tulpule A, Asthana S, Blakely CM, Okimoto RA, Lin L, Neel DS, Sabnis A, Flanagan J, Chan E, Varella-Garcia M, Aisner DL, Vaishnavi A, Ou SH (2015). RAS-MAPK dependence underlies a rational polytherapy strategy in EML4-ALK-positive lung cancer. Nature medicine.

[75] Sharma SV, Settleman J (2007). Oncogene addiction: setting the stage for molecularly targeted cancer therapy. Genes & development.

[76] Ou SH, Soo RA, Kubo A, Kawaguchi T, Ahn MJ (2014). Will the Requirement by the US FDA to Simultaneously Co-Develop Companion Diagnostics (CDx) Delay the Approval of Receptor Tyrosine Kinase Inhibitors for RTK-Rearranged (ROS1-, RET-, AXL-, PDGFR-alpha-, NTRK1-) Non-Small Cell Lung Cancer Globally?. Front Oncol.

[77] Rikova K, Guo A, Zeng Q, Possemato A, Yu J, Haack H, Nardone J, Lee K, Reeves C, Li Y, Hu Y, Tan Z, Stokes M, Sullivan L, Mitchell J, Wetzel R (2007). Global survey of phosphotyrosine signaling identifies oncogenic kinases in lung cancer. Cell.

[78] Gu TL, Deng X, Huang F, Tucker M, Crosby K, Rimkunas V, Wang Y, Deng G, Zhu L, Tan Z, Hu Y, Wu C, Nardone J, MacNeill J, Ren J, Reeves C (2011). Survey of tyrosine kinase signaling reveals ROS kinase fusions in human cholangiocarcinoma. PLoS One.

[79] Cooper MJ, Cox NJ, Zimmerman EI, Dewar BJ, Duncan JS, Whittle MC, Nguyen TA, Jones LS, Ghose Roy S, Smalley DM, Kuan PF, Richards KL, Christopherson RI, Jin J, Frye SV, Johnson GL (2013). Application of multiplexed kinase inhibitor beads to study kinome adaptations in drug-resistant leukemia. PLoS One.

[80] Yanagisawa K, Tomida S, Shimada Y, Yatabe Y, Mitsudomi T, Takahashi T (2007). A 25-signal proteomic signature and outcome for patients with resected non-small-cell lung cancer. J Natl Cancer Inst.

[81] Wu G, Diaz AK, Paugh BS, Rankin SL, Ju B, Li Y, Zhu X, Qu C, Chen X, Zhang J, Easton J, Edmonson M, Ma X, Lu C, Nagahawatte P, Hedlund E (2014). The genomic landscape of diffuse intrinsic pontine glioma and pediatric non-brainstem high-grade glioma. Nature genetics.

[82] Frattini V, Trifonov V, Chan JM, Castano A, Lia M, Abate F, Keir ST, Ji AX, Zoppoli P, Niola F, Danussi C, Dolgalev I, Porrati P, Pellegatta S, Heguy A, Gupta G (2013). The integrated landscape of driver genomic alterations in glioblastoma. Nature genetics.

[83] Seshagiri S, Stawiski EW, Durinck S, Modrusan Z, Storm EE, Conboy CB, Chaudhuri S, Guan Y, Janakiraman V, Jaiswal BS, Guillory J, Ha C, Dijkgraaf GJ, Stinson J, Gnad F, Huntley MA (2012). Recurrent R-spondin fusions in colon cancer. Nature.

[84] Ricarte-Filho JC, Li S, Garcia-Rendueles ME, Montero-Conde C, Voza F, Knauf JA, Heguy A, Viale A, Bogdanova T, Thomas GA, Mason CE, Fagin JA (2013). Identification of kinase fusion oncogenes in post-Chernobyl radiation-induced thyroid cancers. J Clin Invest.

[85] Semrau S, Crosetto N, Bienko M, Boni M, Bernasconi P, Chiarle R, van Oudenaarden A (2014). FuseFISH: robust detection of transcribed gene fusions in single cells. Cell Rep.

[86] Eyal E, Tohami T, Amir A, Cesarkas K, Jacob-Hirsch J, Volchek Y, Nagler A, Rechavi G, Amariglio N (2013). Detection of BCR-ABL1 mutations in chronic myeloid leukaemia by massive parallel sequencing. Br J Haematol.

[87] Sholl LM, Sun H, Butaney M, Zhang C, Lee C, Janne PA, Rodig SJ (2013). ROS1 immunohistochemistry for detection of ROS1-rearranged lung adenocarcinomas. Am J Surg Pathol.

[88] Lira ME, Choi YL, Lim SM, Deng S, Huang D, Ozeck M, Han J, Jeong JY, Shim HS, Cho BC, Kim J, Ahn MJ, Mao M (2014). A single-tube multiplexed assay for detecting ALK, ROS1, and RET fusions in lung cancer. J Mol Diagn.

[89] Santos FP, Kantarjian H, Quintas-Cardama A, Cortes J (2011). Evolution of therapies for chronic myelogenous leukemia. Cancer J.

[90] Shaw AT, Ou SH, Bang YJ, Camidge DR, Solomon BJ, Salgia R, Riely GJ, Varella-Garcia M, Shapiro GI, Costa DB, Doebele RC, Le LP, Zheng Z, Tan W, Stephenson P, Shreeve SM (2014). Crizotinib in ROS1-Rearranged Non-Small-Cell Lung Cancer. N Engl J Med.

[91] Chang BH, Willis SG, Stork L, Hunger SP, Carroll WL, Camitta BM, Winick NJ, Druker BJ, Schultz KR (2012). Imatinib resistant BCR-ABL1 mutations at relapse in children with Ph+ ALL: a Children's Oncology Group (COG) study. Br J Haematol.

[92] Chu S, Li L, Singh H, Bhatia R (2007). BCR-tyrosine 177 plays an essential role in Ras and Akt activation and in human hematopoietic progenitor transformation in chronic myelogenous leukemia. Cancer Res.

[93] Druker BJ, Guilhot F, O'Brien SG, Gathmann I, Kantarjian H, Gattermann N, Deininger MW, Silver RT, Goldman JM, Stone RM, Cervantes F, Hochhaus A, Powell BL, Gabrilove JL, Rousselot P, Reiffers J (2006). Five-year follow-up of patients receiving imatinib for chronic myeloid leukemia. N Engl J Med.

[94] Gambacorti-Passerini C, Antolini L, Mahon FX, Guilhot F, Deininger M, Fava C, Nagler A, Della Casa CM, Morra E, Abruzzese E, D'Emilio A, Stagno F, le Coutre P, Hurtado-Monroy R, Santini V, Martino B (2011). Multicenter independent assessment of outcomes in chronic myeloid leukemia patients treated with imatinib. J Natl Cancer Inst.

[95] Khoury HJ, Cortes JE, Kantarjian HM, Gambacorti-Passerini C, Baccarani M, Kim DW, Zaritskey A, Countouriotis A, Besson N, Leip E, Kelly V, Brummendorf TH (2012). Bosutinib is active in chronic phase chronic myeloid leukemia after imatinib and dasatinib and/or nilotinib therapy failure. Blood.

[96] Chan WW, Wise SC, Kaufman MD, Ahn YM, Ensinger CL, Haack T, Hood MM, Jones J, Lord JW, Lu WP, Miller D, Patt WC, Smith BD, Petillo PA, Rutkoski TJ, Telikepalli H (2011). Conformational control inhibition of the BCR-ABL1 tyrosine kinase, including the gatekeeper T315I mutant, by the switch-control inhibitor DCC-2036. Cancer Cell.

[97] Cortes JE, Kim DW, Pinilla-Ibarz J, le Coutre P, Paquette R, Chuah C, Nicolini FE, Apperley JF, Khoury HJ, Talpaz M, DiPersio J, DeAngelo DJ, Abruzzese E, Rea D, Baccarani M, Muller MC (2013). A phase 2 trial of ponatinib in Philadelphia chromosome-positive leukemias. N Engl J Med.

[98] Pemovska T, Johnson E, Kontro M, Repasky GA, Chen J, Wells P, Cronin CN, McTigue M, Kallioniemi O, Porkka K, Murray BW, Wennerberg K (2015). Axitinib effectively inhibits BCR-ABL1(T315I) with a distinct binding conformation. Nature.

[99] O'Hare T, Zabriskie MS, Eiring AM, Deininger MW (2012). Pushing the limits of targeted therapy in chronic myeloid leukaemia. Nature reviews Cancer.

[100] Eiring AM, Page BD, Kraft IL, Mason CC, Vellore NA, Resetca D, Zabriskie MS, Zhang TY, Khorashad JS, Engar AJ, Reynolds KR, Anderson DJ, Senina A, Pomicter AD, Arpin CC, Ahmad S (2015). Combined STAT3 and BCR-ABL1 inhibition induces synthetic lethality in therapy-resistant chronic myeloid leukemia. Leukemia.

[101] Prost S, Relouzat F, Spentchian M, Ouzegdouh Y, Saliba J, Massonnet G, Beressi JP, Verhoeyen E, Raggueneau V, Maneglier B, Castaigne S, Chomienne C, Chretien S, Rousselot P, Leboulch P (2015). Erosion of the chronic myeloid leukaemia stem cell pool by PPARgamma agonists. Nature.

[102] Jones D, Thomas D, Yin CC, O'Brien S, Cortes JE, Jabbour E, Breeden M, Giles FJ, Zhao W, Kantarjian HM (2008). Kinase domain point mutations in Philadelphia chromosome-positive acute lymphoblastic leukemia emerge after therapy with BCR-ABL kinase inhibitors. Cancer.

[103] Mullighan CG, Williams RT, Downing JR, Sherr CJ (2008). Failure of CDKN2A/B (INK4A/B-ARF)-mediated tumor suppression and resistance to targeted therapy in acute lymphoblastic leukemia induced by BCR-ABL. Genes & development.

[104] Schultz KR, Carroll A, Heerema NA, Bowman WP, Aledo A, Slayton WB, Sather H, Devidas M, Zheng HW, Davies SM, Gaynon PS, Trigg M, Rutledge R, Jorstad D, Winick N, Borowitz MJ (2014). Long-term follow-up of imatinib in pediatric Philadelphia chromosome-positive acute lymphoblastic leukemia: Children's Oncology Group study AALL0031. Leukemia.

[105] Biondi A, Schrappe M, De Lorenzo P, Castor A, Lucchini G, Gandemer V, Pieters R, Stary J, Escherich G, Campbell M, Li CK, Vora A, Arico M, Rottgers S, Saha V, Valsecchi MG (2012). Imatinib after induction for treatment of children and adolescents with Philadelphia-chromosome-positive acute lymphoblastic leukaemia (EsPhALL): a randomised, open-label, intergroup study. Lancet Oncol.

[106] Daver N, Thomas D, Ravandi F, Cortes J, Garris R, Jabbour E, Garcia-Manero G, Borthakur G, Kadia T, Rytting M, Konopleva M, Kantarjian H, O'Brien S (2015). Final report of a phase II study of imatinib mesylate with hyper-CVAD for the front-line treatment of adult patients with Philadelphia chromosome-positive acute lymphoblastic leukemia. Haematologica.

[107] Ravandi F, O'Brien SM, Cortes JE, Thomas DM, Garris R, Faderl S, Burger JA, Rytting ME, Ferrajoli A, Wierda WG, Verstovsek S, Champlin R, Kebriaei P, McCue DA, Huang X, Jabbour E (2015). Long-term follow-up of a phase 2 study of chemotherapy plus dasatinib for the initial treatment of patients with Philadelphia chromosome-positive acute lymphoblastic leukemia. Cancer.

[108] Foa R, Vitale A, Vignetti M, Meloni G, Guarini A, De Propris MS, Elia L, Paoloni F, Fazi P, Cimino G, Nobile F, Ferrara F, Castagnola C, Sica S, Leoni P, Zuffa E (2011). Dasatinib as first-line treatment for adult patients with Philadelphia chromosome-positive acute lymphoblastic leukemia. Blood.

[109] Chiaretti S, Foa R (2015). Management of adult Ph-positive acute lymphoblastic leukemia. Hematology / the Education Program of the American Society of Hematology American Society of Hematology Education Program.

[110] Kwak EL, Bang YJ, Camidge DR, Shaw AT, Solomon B, Maki RG, Ou SH, Dezube BJ, Janne PA, Costa DB, Varella-Garcia M, Kim WH, Lynch TJ, Fidias P, Stubbs H, Engelman JA (2010). Anaplastic lymphoma kinase inhibition in non-small-cell lung cancer. N Engl J Med.

[111] Drilon A, Wang L, Hasanovic A, Suehara Y, Lipson D, Stephens P, Ross J, Miller V, Ginsberg M, Zakowski MF, Kris MG, Ladanyi M, Rizvi N (2013). Response to Cabozantinib in patients with RET fusion-positive lung adenocarcinomas. Cancer Discov.

[112] Solomon B, Wilner KD, Shaw AT (2014). Current status of targeted therapy for anaplastic lymphoma kinase-rearranged non-small cell lung cancer. Clin Pharmacol Ther.

[113] Katayama R, Kobayashi Y, Friboulet L, Lockerman EL, Koike S, Shaw AT, Engelman JA, Fujita N (2015). Cabozantinib overcomes crizotinib resistance in ROS1 fusion-positive cancer. Clinical cancer research.

[114] Tibaldi C (2014). Mechanisms of resistance to crizotinib in patients with ALK gene rearranged non-small-cell lung cancer. Pharmacogenomics.

[115] Zou HY, Li Q, Engstrom LD, West M, Appleman V, Wong KA, McTigue M, Deng YL, Liu W, Brooun A, Timofeevski S, McDonnell SR, Jiang P, Falk MD, Lappin PB, Affolter T (2015). PF-06463922 is a potent and selective next-generation ROS1/ALK inhibitor capable of blocking crizotinib-resistant ROS1 mutations. Proc Natl Acad Sci U S A.

[116] Friedman R (2013). Drug resistance missense mutations in cancer are subject to evolutionary constraints. PLoS One.

[117] Tanizaki J, Okamoto I, Okabe T, Sakai K, Tanaka K, Hayashi H, Kaneda H, Takezawa K, Kuwata K, Yamaguchi H, Hatashita E, Nishio K, Nakagawa K (2012). Activation of HER family signaling as a mechanism of acquired resistance to ALK inhibitors in EML4-ALK-positive non-small cell lung cancer. Clinical cancer research.

[118] Yamada T, Takeuchi S, Nakade J, Kita K, Nakagawa T, Nanjo S, Nakamura T, Matsumoto K, Soda M, Mano H, Uenaka T, Yano S (2012). Paracrine receptor activation by microenvironment triggers bypass survival signals and ALK inhibitor resistance in EML4-ALK lung cancer cells. Clinical cancer research.

[119] Voena C, Di Giacomo F, Panizza E, D'Amico L, Boccalatte FE, Pellegrino E, Todaro M, Recupero D, Tabbo F, Ambrogio C, Martinengo C, Bonello L, Pulito R, Hamm J, Chiarle R, Cheng M (2013). The EGFR family members sustain the neoplastic phenotype of ALK+ lung adenocarcinoma via EGR1. Oncogenesis.

[120] Katayama R, Shaw AT, Khan TM, Mino-Kenudson M, Solomon BJ, Halmos B, Jessop NA, Wain JC, Yeo AT, Benes C, Drew L, Saeh JC, Crosby K, Sequist LV, Iafrate AJ, Engelman JA (2012). Mechanisms of acquired crizotinib resistance in ALK-rearranged lung Cancers. Sci Transl Med.

[121] Wilson FH, Johannessen CM, Piccioni F, Tamayo P, Kim JW, Van Allen EM, Corsello SM, Capelletti M, Calles A, Butaney M, Sharifnia T, Gabriel SB, Mesirov JP, Hahn WC, Engelman JA, Meyerson M (2015). A functional landscape of resistance to ALK inhibition in lung cancer. Cancer Cell.

[122] Pennacchietti S, Cazzanti M, Bertotti A, Rideout WM, Han M, Gyuris J, Perera T, Comoglio PM, Trusolino L, Michieli P (2014). Microenvironment-derived HGF overcomes genetically determined sensitivity to anti-MET drugs. Cancer Res.

[123] Crescenzo R, Inghirami G (2015). Anaplastic lymphoma kinase inhibitors. Current opinion in pharmacology.

[124] Matsuoka H, Kurata T, Okamoto I, Kaneda H, Tanaka K, Nakagawa K (2013). Clinical response to crizotinib retreatment after acquisition of drug resistance. J Clin Oncol.

[125] Chmielecki J, Foo J, Oxnard GR, Hutchinson K, Ohashi K, Somwar R, Wang L, Amato KR, Arcila M, Sos ML, Socci ND, Viale A, de Stanchina E, Ginsberg MS, Thomas RK, Kris MG (2011). Optimization of dosing for EGFR-mutant non-small cell lung cancer with evolutionary cancer modeling. Sci Transl Med.

[126] Voena C, Menotti M, Mastini C, Di Giacomo F, Longo DL, Castella B, Merlo ME, Ambrogio C, Wang Q, Minero VG, Poggio T, Martinengo C, D'Amico L, Panizza E, Mologni L, Cavallo F (2015). Efficacy of a Cancer Vaccine against ALK-Rearranged Lung Tumors. Cancer immunology research.

[127] Subbiah V, Berry J, Roxas M, Guha-Thakurta N, Subbiah IM, Ali SM, McMahon C, Miller V, Cascone T, Pai S, Tang Z, Heymach JV (2015). Systemic and CNS activity of the RET inhibitor vandetanib combined with the mTOR inhibitor everolimus in KIF5B-RET re-arranged non-small cell lung cancer with brain metastases. Lung Cancer.

[128] Perez-Soler R, Chachoua A, Hammond LA, Rowinsky EK, Huberman M, Karp D, Rigas J, Clark GM, Santabarbara P, Bonomi P (2004). Determinants of tumor response and survival with erlotinib in patients with non—small-cell lung cancer. J Clin Oncol.

[129] Benjamini O, Dumlao TL, Kantarjian H, O'Brien S, Garcia-Manero G, Faderl S, Jorgensen J, Luthra R, Garris R, Thomas D, Kebriaei P, Champlin R, Jabbour E, Burger J, Cortes J, Ravandi F (2014). Phase II trial of hyper CVAD and dasatinib in patients with relapsed Philadelphia chromosome positive acute lymphoblastic leukemia or blast phase chronic myeloid leukemia. American journal of hematology.

[130] Harvey RD (2014). Immunologic and clinical effects of targeting PD-1 in lung cancer. Clin Pharmacol Ther.

[131] Benyamini N, Rowe JM (2013). Is there a role for allogeneic transplantation in chronic myeloid leukemia?. Expert Rev Hematol.

[132] Zhai H, Zhong W, Yang X, Wu YL (2015). Neoadjuvant and adjuvant epidermal growth factor receptor tyrosine kinase inhibitor (EGFR-TKI) therapy for lung cancer. Translational lung cancer research.

[133] Xiao BK, Yang JY, Dong JX, Ji ZS, Si HY, Wang WL, Huang RQ (2015). Meta-analysis of seven randomized control trials to assess the efficacy and toxicity of combining EGFR-TKI with chemotherapy for patients with advanced NSCLC who failed first-line treatment. Asian Pacific journal of cancer prevention.

[134] Das A, Cheng RR, Hilbert ML, Dixon-Moh YN, Decandio M, Vandergrift WA, Banik NL, Lindhorst SM, Cachia D, Varma AK, Patel SJ, Giglio P (2015). Synergistic Effects of Crizotinib and Temozolomide in Experimental FIG-ROS1 Fusion-Positive Glioblastoma. Cancer growth and metastasis.

[135] Appelmann I, Rillahan CD, de Stanchina E, Carbonetti G, Chen C, Lowe SW, Sherr CJ (2015). Janus kinase inhibition by ruxolitinib extends dasatinib- and dexamethasone-induced remissions in a mouse model of Ph+ ALL. Blood.

[136] Leder K, Pitter K, Laplant Q, Hambardzumyan D, Ross BD, Chan TA, Holland EC, Michor F (2014). Mathematical modeling of PDGF-driven glioblastoma reveals optimized radiation dosing schedules. Cell.

[137] Aparicio S, Hidalgo M, Kung AL (2015). Examining the utility of patient-derived xenograft mouse models. Nature reviews Cancer.

[138] Bertotti A, Papp E, Jones S, Adleff V, Anagnostou V, Lupo B, Sausen M, Phallen J, Hruban CA, Tokheim C, Niknafs N, Nesselbush M, Lytle K, Sassi F, Cottino F, Migliardi G (2015). The genomic landscape of response to EGFR blockade in colorectal cancer. Nature.

[139] Gao H, Korn JM, Ferretti S, Monahan JE, Wang Y, Singh M, Zhang C, Schnell C, Yang G, Zhang Y, Balbin OA, Barbe S, Cai H, Casey F, Chatterjee S, Chiang DY (2015). High-throughput screening using patient-derived tumor xenografts to predict clinical trial drug response. Nature medicine.

[140] Fang DD, Zhang B, Gu Q, Lira M, Xu Q, Sun H, Qian M, Sheng W, Ozeck M, Wang Z, Zhang C, Chen X, Chen KX, Li J, Chen SH, Christensen J (2014). HIP1-ALK, a novel ALK fusion variant that responds to crizotinib. J Thorac Oncol.

[141] Cheng M, Quail MR, Gingrich DE, Ott GR, Lu L, Wan W, Albom MS, Angeles TS, Aimone LD, Cristofani F, Machiorlatti R, Abele C, Ator MA, Dorsey BD, Inghirami G, Ruggeri B (2011). CEP-28122, a Highly Potent and Selective Orally Active Inhibitor of Anaplastic Lymphoma Kinase with Antitumor Activity in Experimental Models of Human Cancers. Mol Cancer Ther.

[142] Dong X, Jin K, Hu X, Du F, Lan H, Han N, Ma Z, Xie B, Cui B, Teng L, Cao F (2012). Antitumor effect of FP3 in combination with cetuximab on patient-derived tumor tissue xenograft models of primary colon carcinoma and related lymphatic and hepatic metastases. International journal of molecular medicine.

